# Speed Controls the Amplitude and Timing of the Hippocampal Gamma Rhythm

**DOI:** 10.1371/journal.pone.0021408

**Published:** 2011-06-24

**Authors:** Zhiping Chen, Evgeny Resnik, James M. McFarland, Bert Sakmann, Mayank R. Mehta

**Affiliations:** 1 Department of Physics and Astronomy, and Integrative Center for Learning and Memory, University of California at Los Angeles, Los Angeles, California, United States of America; 2 Department of Cell Physiology, Max-Planck-Institute for Medical Research, Heidelberg, Germany; 3 Department of Physics, Brown University, Providence, Rhode Island, United States of America; 4 Max-Plank-Florida Institute, Jupiter, Florida, United States of America; 5 Departments of Neurology and of Neurobiology, University of California at Los Angeles, Los Angeles, California, United States of America; Max-Planck Institute of Neurobiology, Germany

## Abstract

Cortical and hippocampal gamma oscillations have been implicated in many behavioral tasks. The hippocampus is required for spatial navigation where animals run at varying speeds. Hence we tested the hypothesis that the gamma rhythm could encode the running speed of mice. We found that the amplitude of slow (20–45 Hz) and fast (45–120 Hz) gamma rhythms in the hippocampal local field potential (LFP) increased with running speed. The speed-dependence of gamma amplitude was restricted to a narrow range of theta phases where gamma amplitude was maximal, called the preferred theta phase of gamma. The preferred phase of slow gamma precessed to lower values with increasing running speed. While maximal fast and slow gamma occurred at coincident phases of theta at low speeds, they became progressively more theta-phase separated with increasing speed. These results demonstrate a novel influence of speed on the amplitude and timing of the hippocampal gamma rhythm which could contribute to learning of temporal sequences and navigation.

## Introduction

Gamma rhythmic (∼20–120 Hz) modulation of neural activity has been demonstrated in the cortex [1], [2], [3], [4], [5], [6], [7], [8], [9] and hippocampus [10], [11], [12], [13], [14], [15]. Gamma oscillations are thought to increase synchronization of neural activity to mediate a variety of cognitive functions including attention [4], learning [14], temporal binding and awareness [9], [16]. Gamma oscillations in the hippocampal LFP of rats are also modulated by theta oscillations, and they occur in two distinct bands, the lower frequency slow gamma (30–55 Hz) and the higher frequency fast gamma (55–120 Hz). The slow and fast gamma oscillations in CA1 are synchronous with slow gamma in CA3 and fast gamma in the entorhinal cortex respectively [15]. Gamma rhythm also separates into the slow and fast bands in the human sensorymotor cortex [3] and the rodent olfactory bulb [7].

Gamma oscillations in CA1 are larger in mice than in rats [11]. Thus, mouse hippocampal gamma provides a reliable way of studying its modulation by behavior. In behaving mice, the hippocampal gamma rhythm and its coupling to theta are influenced by parvalbumin containing interneurons [17], interneuron-interneuron gap junctions [11], [18], and acetylcholine [19], thereby implicating complex interactions between cellular properties, the excitatory and inhibitory neuronal networks and neuromodulators. The contribution of gamma oscillations to navigation is unknown.

During spatial navigation hippocampal pyramidal neurons, or place cells, fire at elevated rates in restricted regions of space [20], thereby providing information about the subject's position through a rate code [21]. The pyramidal neurons' activity also provides information about a rat's position through a temporal code known as theta phase precession such that the phase of the LFP theta rhythm where a pyramidal neuron spikes systematically precesses to lower values as a function of the position of the rat [22], [23], [24], [25], [26], [27].

In order to navigate, it is not only necessary to know the current position but also to predict the future position. A necessary condition to achieve this is to know the current speed, in addition to knowing position and head direction information. Firing rates of place cells contain information about position and head direction [28]. In addition, place cells' firing rates [28], [29], and hippocampal interneuron firing rates [30] also increase with speed. However, since position and speed are orthogonal variables, using firing rates to encode both position and speed could be confounding. We hypothesize that hippocampal high frequency oscillations could provide an independent and fast code for running speed.

Here we show that the amplitude of the gamma rhythm is strongly modulated by running speed. Unlike previously reported abrupt changes in gamma with task variables, we report a gradual increase in gamma amplitude with running speed that differentially influences slow and fast gamma rhythms. Further, we demonstrate a theta-phase precession like phenomenon where the preferred theta-phase of the slow gamma rhythm precesses to lower values with increasing running speed of the mouse.

## Results

**Figure 1:**
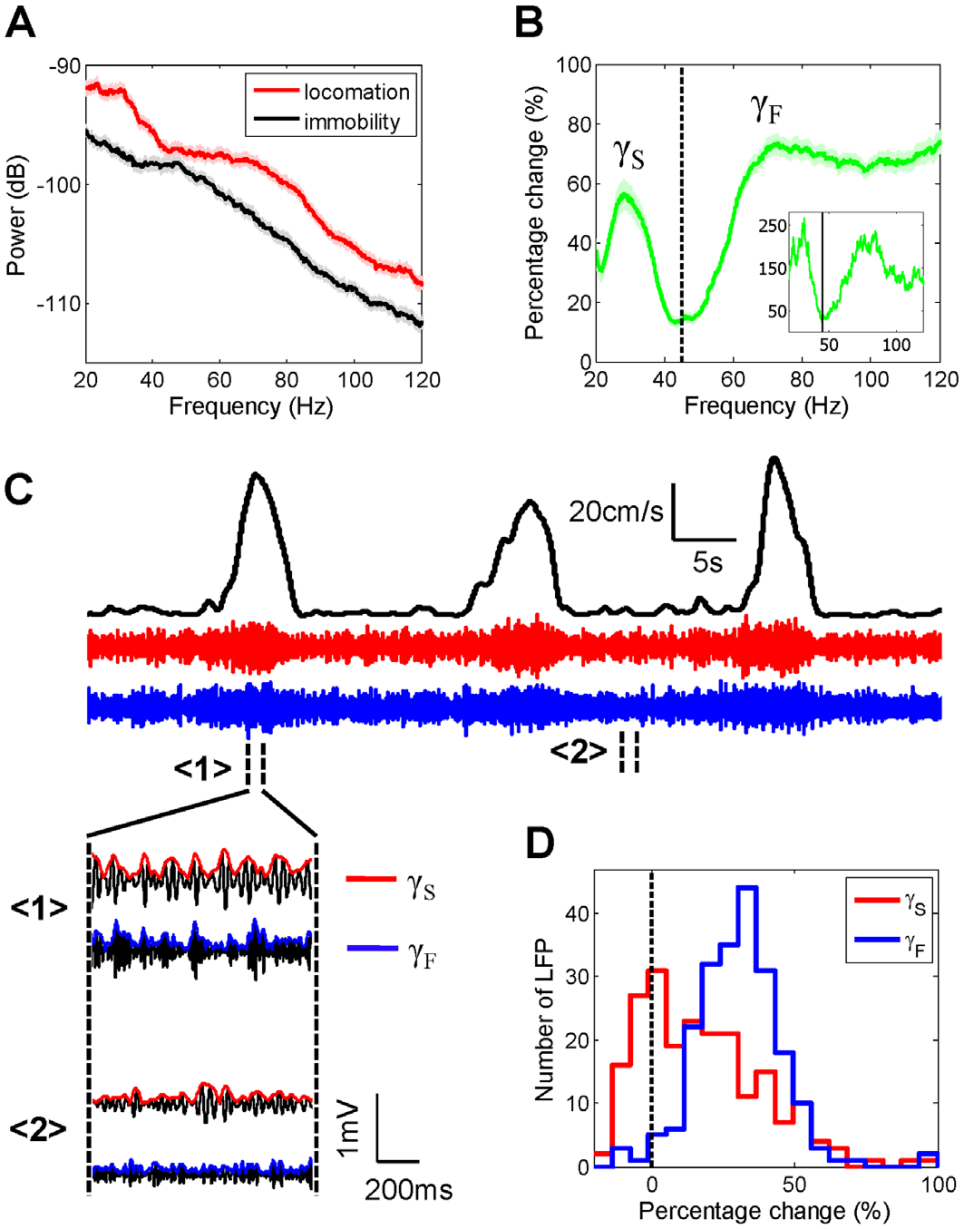
Hippocampal gamma rhythm splits into two bands, fast and slow, whose magnitude increases during locomotion. **A**) Power spectrum of a dorsal hippocampal LFP during locomotion (red) and immobility (black) in one example session. Shaded areas indicate 95% confidence intervals. **B**) Change in spectral power during run compared to stop as a function of frequency in the gamma band. Data are averaged across the ensemble of 214 LFP traces. Shaded regions correspond to s.e.m. here and in subsequent figures. Inset shows the change in power for the example data set from fig. 1a. The increase in power is significantly lower at 45 Hz than in the surrounding frequency band, thereby demarcating a clear border between slow (20–45 Hz) and fast (45–120 Hz) gamma bands. **C**) Running speed of a mouse as a function of time (black), and corresponding amplitude of slow (red) and fast (blue) gamma rhythms. Insets show slow and fast gamma amplitudes at higher temporal resolution for high (<1>) and low (<2>) speeds. Both fast and slow gamma amplitudes are larger during run than stop. **D**) Slow (red) and fast (blue) gamma amplitudes were 15±1.5% (p = 5.3e-24) (mean±s.e.m., Wilcoxon Ranksum test here and in subsequent figures), and 31±1.0% (p = 1.2e-65) larger during run than stop, with fast gamma amplitude showing a greater increase than slow gamma (p = 1.5e-13).

We measured 214 LFPs along with spiking activity, from the dorsal hippocampal area CA1 in 63 sessions from twelve mice using tetrodes. The mice ran on a 1.5 m long linear track to obtain rewards at the two ends of the track (see Methods S1). The only selection criteria for using the data were that the tetrode was in the hippocampus while the mouse ran on the linear track. For the analysis of spiking activity, only those (160) tetrodes with at least 500 spikes on the track were used to ensure reliable quantification. As is common, it was difficult to detect a clear boundary between the fast and slow gamma bands based on the spectral power of the LFP in the gamma range alone (figure 1a, the same data were used for all subsequent example figures). This could result from noise masking a potential boundary between the gamma sub-bands. To overcome this difficulty, we assumed that the noise would remain relatively unchanged between the stop and run epochs, and computed the percentage change in spectral power at each frequency during run relative to the corresponding power during immobility. This procedure not only showed a clear increase in power in the gamma range during locomotion, it also revealed the change in gamma power was bimodal, clearly separating in two sub-bands within the gamma range (figure 1b, see methods): slow gamma (20–45 Hz) and fast gamma (45–120 Hz). The boundary between the two gamma bands was defined as the frequency (45 Hz) at which there was the smallest change in gamma power between run and stop. This split in the gamma band is similar to studies in rats [10], [13], [15], but the entire gamma band is ∼10 Hz lower in our data in mice.

Having detected the boundary between the two gamma bands, all subsequent analyses were done using the raw LFP, without any subtraction. The instantaneous amplitudes of the LFP were calculated separately for the slow (20–45 Hz) and fast (45–120 Hz) gamma bands, in order to obtain greater temporal precision in our estimates of gamma dynamics (figure 1c, Methods S1). The amplitude of both slow and fast gamma rhythms increased by 15±1.5%, p = 5.3e-24 and 31±1.0% p = 1.2e-65 (median±s.e.m., Wilcoxon rank sum test, here and in all data) during run compared to immobility respectively (figure 1d, supplement S1). Thus, there was a significant increase in the amplitude of slow and fast gamma rhythms during locomotion compared to immobility.

**Figure 2:**
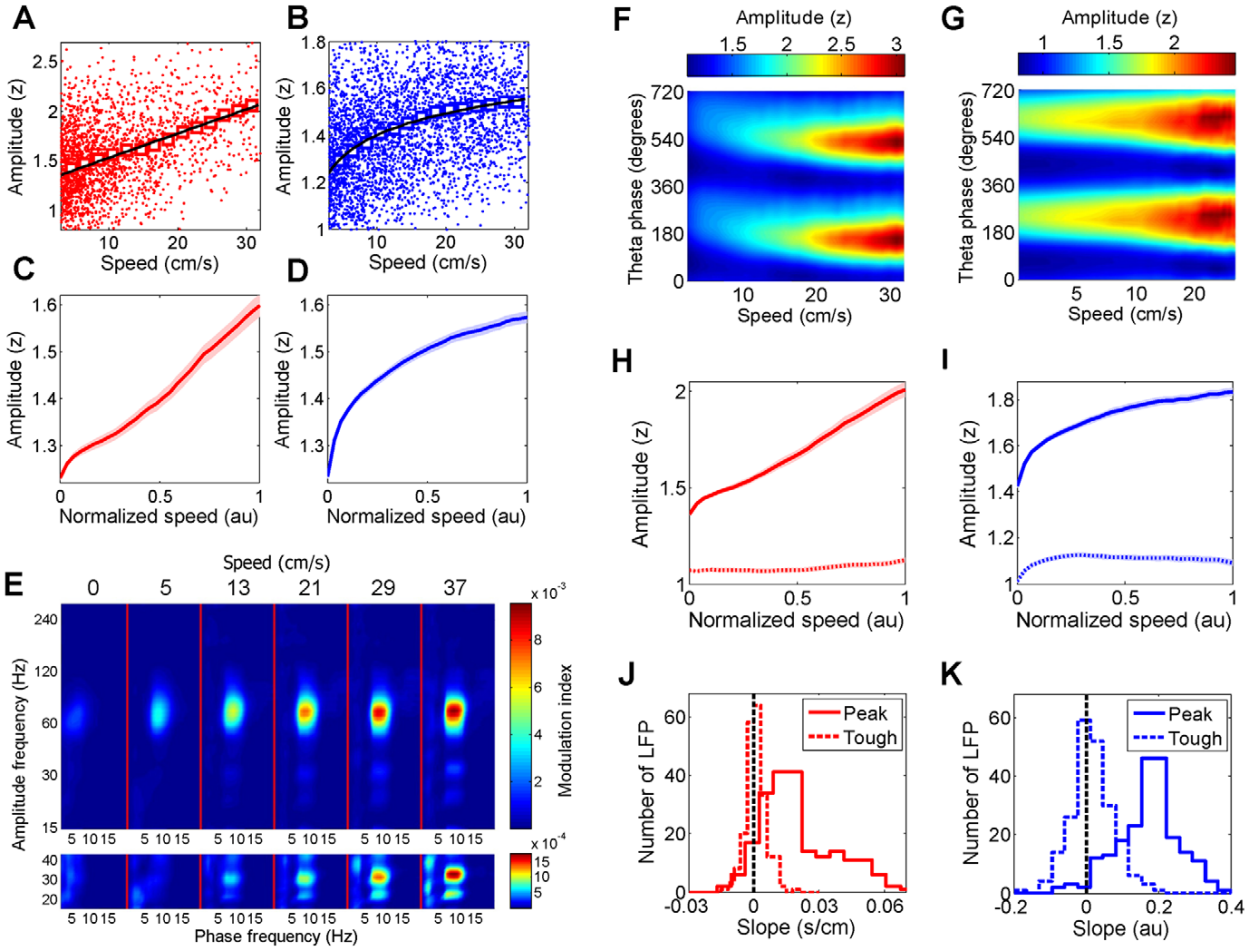
Joint influence of running speed and theta phase on gamma amplitude. **A**) Each red dot depicts the amplitude of slow gamma in a window of 250 ms around each LFP theta peak as a function of running speed. The value of the slow gamma amplitude was averaged within a given speed bin (∼7 cm/s wide, with 80% overlap between neighboring bins) (red squares). Black line shows the best linear fit. **B**) Same as A for fast gamma (blue dots and squares) with logarithmic fit. (See Methods S1 for methods and Supplement S1, Supplement S2 for details). **C**) Ensemble averaged data showing linear increases in slow gamma amplitude with speed. **D**) Same as C with logarithmic speed-dependence for fast gamma. **E**) Each vertical panel shows the cross-frequency coupling between the amplitude of a fast (15–300 Hz) signal (y-axis) and the phase of a slow (2–20 Hz) signal, whose frequency is shown on the x-axis). Separate panels show coupling at different running speeds (top) for the example data in figures 2A,B. Colorbar to the right indicates modulation index (see Methods S1). Significant cross-frequency coupling is found only between the phase of the theta (6–12 Hz) oscillation and the gamma amplitude (20–120 Hz). Fast-gamma-theta coupling is greater than slow-gamma-theta coupling (bottom panel) at all speeds. The coupling increases logarithmically and linearly with speed for fast and slow gamma respectively (see Supplement S2). **F**) Slow gamma amplitude changes with running speed and theta phase for the example data set in figure 2A,B. **G**) Similarly for fast gamma. **H**) Slow gamma amplitude at the preferred phase (at 236±2.2°) of theta, averaged across all data, is linearly correlated with running speed (solid line, R^2^ = 0.90±0.018, median±s.e.m.), but slow gamma amplitude around the theta trough changed minimally (dotted line). **I**) Similarly, fast gamma amplitude around the peak (260±1.8°) of theta increased logarithmically with speed (solid line, R^2^ = 0.94±0.016), but fast gamma amplitude around the theta trough changed minimally (dotted line). **J**) Distribution of the slope of slow gamma amplitude around the theta peak as a function of running speed (solid line) across the ensemble of data, showing that it was significantly positive (0.017±0.0012, p = 1.9e-40) and far greater than the slope around the theta-trough (dotted line, 0.00067±0.00032, p = 4.3e-4). **K**) Similar results were true for the slope of fast gamma amplitude as a function of the logarithm of running speed around the theta peak (solid line, 0.17±0.0057, p = 4.1e-68) and the theta trough (0.013±0.0040, p = 0.0054).

To compare data obtained from different electrodes and mice, the gamma band amplitudes were measured in z-scored units (see methods). Not only did the gamma amplitude increase during locomotion, this increase was proportional to running speed (figure 2). While the slow gamma amplitude increased linearly with running speed (figure 2a, c), fast gamma amplitude depended on the logarithm of running speed (figure 2b, d, supplement S2). We denote the slow and fast gamma amplitudes by *A_S_* and *A_F_* respectively, and running speed by S. The dependence of slow and fast gamma on speed can be modeled by:
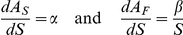
with *α* = 0.017±0.0012 *s/cm* and β = 0.17±0.0057. In other words, since slow gamma amplitude is a linear function of speed, the derivative of slow gamma amplitude with respect to speed is independent of speed. Similarly, since the fast gamma amplitude increases as the logarithm of running speed, the derivative of fast gamma amplitude with respect to speed is inversely proportional to speed. The small magnitude of standard errors in *α* and *β* compared to their mean is indicative of a remarkably consistent fit across datasets. These functions were also very good fits to the data, as confirmed by the analysis of residual errors (less than 5%) of linear and logarithmic fits (supplement S3). Thus, the amplitudes of fast and slow gamma rhythms were differentially, gradually, stochastically but reliably modulated by running speed.

The amplitude of the gamma rhythm is modulated by the phase of a lower frequency LFP theta rhythm in rats [10], [13], [14], [15], mice [31], [32] and humans [8]. Hence, we investigated the speed-dependence of cross-frequency coupling between the phase of low frequency rhythms and the amplitude of higher frequency rhythms (figure 2e, see Methods S1). Briefly, cross-frequency coupling was computed using a modulation index based on Shannon information such that a uniform phase distribution would yield a modulation index of zero. Consistent with theta-gamma coupling reported in rats [10], [13], [14], [15] we found significant theta-gamma coupling in mice. In fact, of all the low (2–20 Hz) and high (15–300 Hz) frequencies tested, the strongest cross-frequency phase-amplitude coupling was found between the phase of the theta rhythm (6–12 Hz) and the amplitudes of the slow and fast gamma rhythms. In addition, theta-gamma coupling was significantly larger for fast compared to slow gamma (figure 2e, Supplement S4). The cross-frequency coupling increased linearly with speed for slow gamma but logarithmically for fast gamma (Supplement S4), as with the amplitudes.

To understand the fine temporal structure of theta-gamma coupling, we computed the joint influence of theta phase and speed on slow (figure 2f) and fast (figure 2g) gamma amplitudes. The trough of theta was designated as phase 0° or 360°. The multiunit firing was maximal around the trough of theta (358°±3.7°, see methods). The phase of theta where gamma amplitude was maximal was called its preferred theta phase (see methods). Both slow and fast gamma had their preferred theta phases in the descending part of theta (figure 2f, g, Supplement S6). Further, only the gamma amplitude around the preferred theta phase showed modulation with speed (figure 2h, i). The slow gamma amplitude around its preferred theta phase showed a 44±2.9% linear increase with speed, whereas slow gamma amplitude 180° away from the preferred phase, corresponding to the anti-preferred phase, showed little speed-dependent increase (2.6±1.1%). Similarly, fast gamma amplitude around its preferred phase showed a 32±1.0%, logarithmic increase with speed, whereas fast gamma amplitude 180° away from its preferred phase increased by only 6.5±1.0%. These results were confirmed independently by comparing the slopes of the dependence of gamma amplitude on speed at their preferred theta phase or 180° away from their preferred theta phase for slow (figure 2j) and fast (figure 2k) gamma respectively.

**Figure 3:**
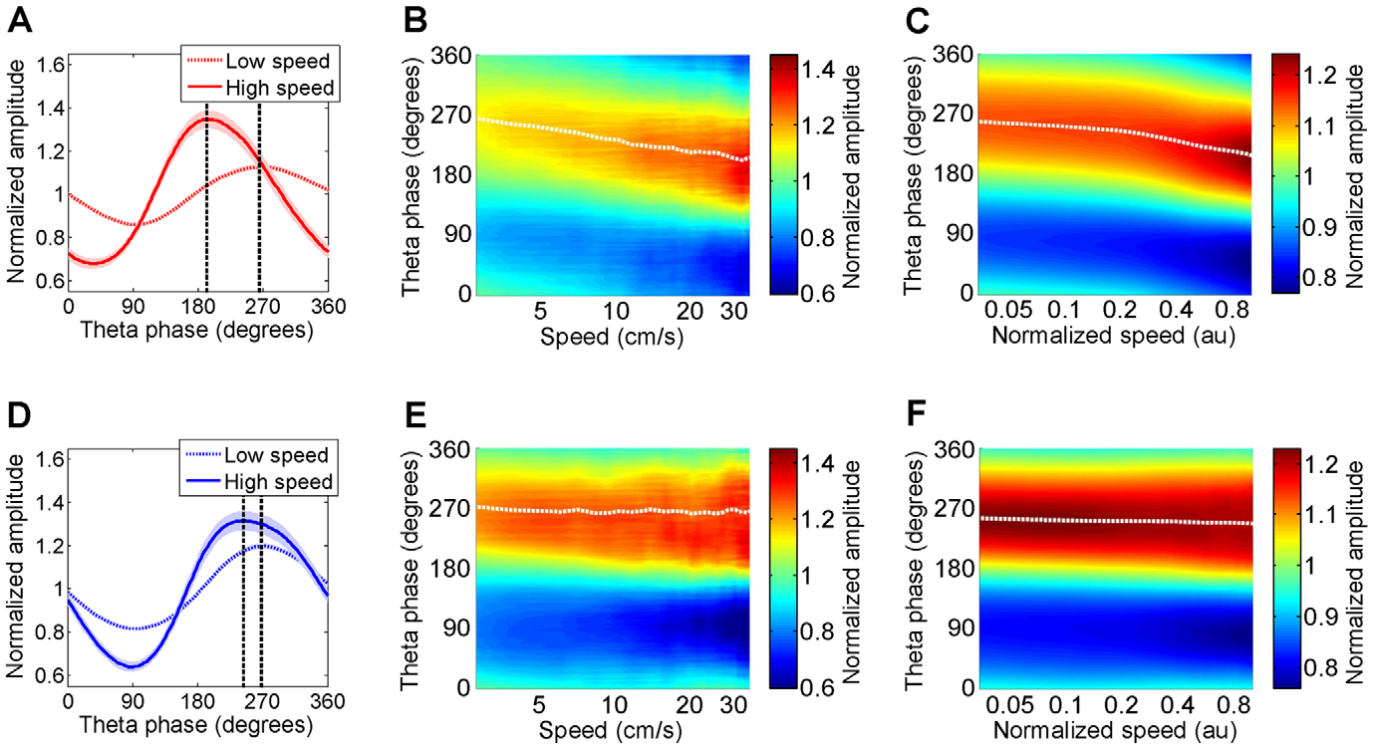
Theta-phase precession of gamma amplitude as a function of running speed. **A**) Slow gamma amplitude as a function of theta phase for one example dataset (figure 2F) was averaged across all the theta cycles at low (dashed) and high (solid) speeds. Further, unlike figure 2F, the amplitude of gamma at each phase of theta was divided by the sum of gamma amplitudes across all phases of theta at that speed. This enabled a comparison of the depth of modulation of gamma amplitude with running speed and theta phase, independent of changes in gamma amplitude with running speed. The theta phase modulation of gamma amplitude was 158% greater and the preferred phase of theta was 63° lower at high speeds compared to low speeds **B**) Same data as A, but as a function of (the logarithm of) a range of running speeds. The hippocampal velo-temporal receptive field for speed (VTRF) for this example dataset shows a progressive precession of slow gamma preferred phase of theta with speed (white dotted line). **C**) VTRF averaged across all the data show a robust increase in the depth of modulation as well as precession (white dotted line) of slow gamma preferred phase of theta with increasing running speed. **D**) Same as in A, but for fast gamma showing only a small change in the depth of modulation of fast gamma amplitude (75%) and preferred theta phase (10.7°) with speed. **E**) Same as B showing only small changes in fast gamma VTRF with speed. **F**) Same as C showing minimal changes in the ensemble averaged fast gamma VTRF with speed.

These findings show that the influence of speed on gamma amplitude was restricted to a narrow range of preferred theta phases. In addition, the preferred theta phase of slow gamma seemed to change with running speed (figure 2f). To demonstrate this visually, it is necessary to suppress the speed-dependent change in gamma amplitude. Dividing the gamma amplitude at all theta phases for a given speed by the gamma amplitude averaged across all the phases in that speed bin achieved this suppression and revealed not only that the depth of modulation of slow gamma amplitude by theta phase was 158% larger at higher speeds, but also that the preferred theta phase of slow gamma was phase-advanced by 63° at high compared to low speeds (figure 3a). This is similar to precession of spike phase as a function of position [22], [23], [24], [25], [26], [27], that is accompanied by increasing firing rate [24], [33] and membrane potential depolarization [27], that is characterized by the hippocampal spatio-temporal receptive field [24], [26].

The gradual precession of slow gamma preferred theta phase with speed can be analogously characterized by a so-called velo-temporal receptive field (VTRF figure 3b, Supplement S5). The VTRF averaged across the ensemble of data showed robust precession of slow gamma preferred theta phase with speed (figure 3c). Although slow gamma amplitude increased linearly with speed, slow gamma preferred theta phase precessed approximately logarithmically with speed. Unlike slow gamma, fast gamma preferred phase showed only a small amount of precession for the example data set (figure 3e), as well as when averaged across the ensemble of data (figure 3f, supplement S5).

**Figure 4:**
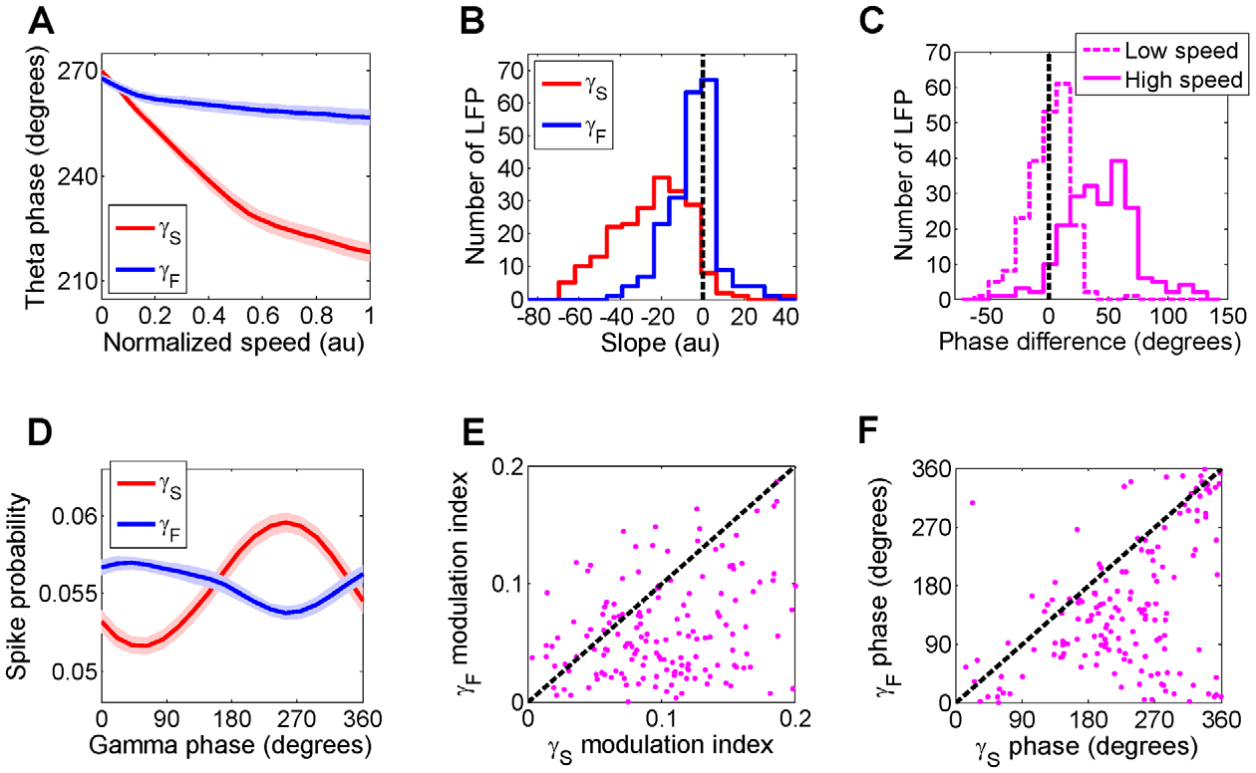
Speed dependent separation of slow and fast gamma preferred phases of theta and modulation of spiking by fast and slow gamma. **A**) Averaged across the ensemble of data, slow and fast gamma preferred theta phases were nearly coincident (−6.0±1.2°) at low speeds, but the slow gamma preferred phase precessed by 61±2.7° with increasing speed whereas fast gamma preferred phase precessed by only 16±1.8°. **B**) The slope of slow (fast) gamma preferred phase as a function of running speed is shown in red (blue). Maximal speed in each session was normalized to unity to allow comparison across data. The vast majority (95%) of slow gamma LFP showed speed-dependent phase advancement, but only 67% of fast gamma LFP showed phase advancement. **C**) There was only a small difference in the preferred phases of slow and fast gamma at low speeds (dashed line, −6.0±1.2°, p = 5.0e-7), but the two rhythms were separated by 37±2.1° (p = 1.3e-25) at high speeds (solid line). **D**) Multi-unit spike probability as a function of fast (blue) and slow (red) gamma phase was computed separately for 141 data sets and averaged across the entire ensemble (see Methods S1). **E**) Scatter plot of fast gamma phase vs. slow gamma modulation index of spiking for 141 data sets. Spike probability was more strongly modulated by the phase of slow gamma than fast gamma (p = 1.9e-14) **F**) Scatter plot of the preferred fast (81±5.7 degrees) and slow gamma phases (242±5.6 degrees) of spikes.

Across the ensemble of data, the preferred theta phase of slow gamma precessed by 61°±2.7° between low and high speeds, whereas the preferred theta phase of fast gamma precessed by only 16°±1.8° (figure 4a, Supplement S7). Thus, while slow and fast gamma rhythms preferred similar phases of theta at low speeds, the two rhythms became increasingly phase-separated with increasing running speed (figure 4a–c). This was further demonstrated by computing the slopes of the slow (and fast) gamma preferred theta phase versus the logarithm of speed. Slopes of gamma preferred theta phase versus speed were negative for both slow (−23±1.4) and fast (−3.0±0.78) gamma, with slow gamma showing significantly greater change with speed than fast gamma (p = 9.14e-34, figure 4b). As a result, the slow and fast gamma preferred theta phases were similar at low speeds, but at the highest speed the slow gamma preferred theta phase occurred 37°±2.1° ahead of the fast gamma preferred phase (figure 4c, Supplement S8). Under laboratory conditions, mice did not attain speeds beyond 50 cm/s. In the wild, mice can attain much higher speeds. Extrapolation of the traces in figure 4a suggests that at these naturally attainable higher speeds, slow and fast gamma can achieve a greater degree of theta-phase separation.

Consistent with previous studies [15], [31], [34], the probability of spiking of the hippocampal neural ensemble, as measured by multi-unit activity, was significantly influenced by the phase of both fast and slow gamma rhythms recorded on the same tetrode (figure 4d, see Methods S1). Additionally, the phase of the large amplitude slow gamma rhythm had a significantly (78.01%, p = 1.9e-14) greater influence on spiking probability than the phase of fast gamma (figure 4d, e, see Methods S1). Further, spikes preferred nearly the opposite phases of slow and fast gamma rhythms [15] (figure 4d, f) with maximal spiking probability occurring at 240°±5.6° of slow gamma (p = 1.7e-7, Rayleigh test) and 80°±5.7° degrees of fast gamma (p = 2.9e-6, Rayleigh test).

## Discussion

These results demonstrate a gradual, large, differential and significant modulation of the amplitude and timing of hippocampal slow and fast gamma oscillations with running speed.

Virtually all of our data show that slow gamma amplitude increases linearly with speed whereas fast gamma amplitude shows a logarithmic dependence on speed (figure 2c, d, Supplements S2, S3). This differential modulation could arise due to mechanisms within MEC and CA3, which are hypothesized to generate the fast and slow gamma respectively [10], [15], or due to the differential nature of excitatory-inhibitory networks and dendritic properties within the distal versus the proximal parts of CA1 where these inputs terminate respectively. Further, cholinergic levels may increase with speed which could differentially enhance gamma oscillations. Speed-dependent changes in gamma rhythm could not be an artifact of speed-dependent change in spiking probability because the multi-unit activity showed far greater phase locking to slow gamma than fast gamma, even though the fast gamma band has more similar frequency content to spikes, thereby increasing the chances of spike bleed over. Further, spikes prefer nearly the opposite phases of slow and fast gamma.

Speed modulation of gamma was restricted to a narrow range of preferred theta phases. The preferred phase of theta, where slow gamma amplitude was maximal, precessed to lower values with increasing speed. This precession of the gamma preferred theta phase was larger for slow than fast gamma. This provides the first demonstration of a phase-precession like phenomenon within the hippocampus that is independent of position and depends on speed. As a result, at low speeds, the highest amplitudes of slow and fast gamma occurred at similar phases of theta, but with increasing speed, they became increasingly more phase-separated such that slow gamma occurred increasingly earlier than fast gamma within each theta cycle.

Further studies are required to determine the biophysical mechanisms underlying these results. One possibility is that with increasing speed, stimuli occur at a greater pace, which increases the firing rates of hippocampal excitatory [28], [29] and inhibitory [30] neurons. This increased spiking activity would facilitate the generation of gamma oscillations in the recurrent excitatory-inhibitory circuits [35] which could explain our finding of gradually increasing gamma amplitude with speed. Since the firing rates of hippocampal neurons are modulated by the phase of the theta rhythm, such a mechanism could also explain the comodulation of gamma amplitude by theta phase and running speed.

Precession of slow gamma preferred theta phase with running speed could be explained by a mechanism similar to the rate-to-phase transformation mechanism proposed to explain theta-phase precession of spikes as a function of position [24], [33], [36], [37] as follows. The firing rate [33] and excitability [27] of place cells increases as a function of the rat's position within the place field. The interaction between this ramping excitation and the theta rhythm could result in theta phase precession of place cell spikes [22], [23], [24], [25], [26], [27]. Similarly, slow gamma oscillations could require a specific balance of excitation and inhibition which occurs at a specific phase (∼270°) of theta at low speeds. The excitation-inhibition balance would be influenced by both theta rhythm and running speed. Higher running speeds could result in increased excitability of neurons which could result in the optimal balance occurring at earlier phases of theta, resulting in slow gamma oscillations appearing at earlier phases of theta, i.e. precession of slow gamma preferred theta phase with speed.

Similar mechanisms could apply to the fast gamma rhythm. However, fast gamma amplitude showed a weaker dependence on speed than slow gamma (figure 2). This could arise due to a smaller speed-dependent increase in firing rates in the entorhinal cortex [38] than the hippocampus [28], [29], or due to local mechanisms within distal regions of CA1 where the entorhinal inputs terminate.

Unlike the theta phase precession of spikes as a function of position, where the spike-phase progressively decreases with increasing position [22], [23], [24], [25], [26], [27], the preferred theta-phase of slow gamma can both increase and decrease with corresponding change in running speed. These observations are consistent with the rate-phase transformation model [24], [33], [36], [37] because position increases only monotonically in the highly directional place cells on linear tracks, whereas speed can change bidirectionally. Consistent with this model, bidirectional changes in preferred gamma phase of spikes have been recently observed [39].

These results can have significant functional consequences. In order to navigate, it is not only necessary to know the current location but also the running speed. Position and speed are orthogonal variables that should be represented by independent parameters. The firing rates of hippocampal neurons are modulated by both spatial location and by the running speed of the animal. Hence, the firing rate provides an ambiguous code for position and speed. Speed-dependent changes in gamma rhythm can provide an independent parameter to encode speed. Just as gamma oscillations can arise through excitatory-inhibitory networks, gamma-rhythmic modulation of spikes can be readily decoded by downstream excitatory-inhibitory networks to extract unambiguous information about speed.

Several studies have shown increased gamma power occurs with attention or task demands, and it is associated with improvement on a number of behavioral measures [4], [9], [14], [16]. Increased gamma power with speed could similarly improve synchrony of hippocampal spikes that could facilitate induction of synaptic plasticity and learning of navigational routes and temporal sequences. It has been suggested that hippocampal activity predicts or anticipates the upcoming events in a temporal sequence [37]. Increasing speed would require faster prediction of future events. If slow and fast gamma arise in CA3 and MEC respectively [10], [15], our results of a speed-dependent increase in the theta phase separation of slow and fast gamma would suggest that CA3 activity increasingly anticipates MEC activity by about 40° of theta phase, or at least 15 ms at high speeds.

Several studies have shown that coincident activation of the entorhinal and CA3 inputs to CA1 results in enhanced activation of CA1 and induction of long-term potentiation of synapses [40], [41], [42], [43], [44]. Thus, at low speeds, coincident activation of CA3 and entorhinal inputs at similar theta phases would increase their efficacy in activating CA1, and facilitate associative synaptic plasticity between these inputs resulting in improved place learning. Increasing theta-phase separation between the slow and fast gamma rhythms at higher speeds would result in anticipatory learning of sequences of neural events between CA3 and MEC inputs within CA1. Such predictive coding is consistent with the observation that stimulation of CA3 before the entorhinal cortex increases the transmission of entorhinal inputs to CA1 neuron's soma [42] by overcoming fast inhibition. Indeed, synchronous activation of entorhinal activity during up-down states results in fast activation of the R-LM interneurons [45] which reduces the level of depolarization of CA1 pyramidal neurons [46]. Thus, the speed-dependent increase in the theta-phase separation of slow and fast-gamma amplitudes could facilitate a speed-dependent enhancement of the efficacy of entorhinal activity, or the sensory inputs, in driving CA1 neurons.

In sum, just as the spiking rates of pyramidal neurons and their preferred theta phases are modulated by spatial position, we show that the magnitude of slow and fast gamma rhythms and their preferred theta phases are modulated by running speed. This suggests that while the firing rate and theta phase of pyramidal neurons encode position, the amplitude and theta phase of gamma oscillations can provide an independent estimate of running speed that could be useful in navigation and learning.

## Materials and Methods

### Ethics statement

All experiments were conducted in accordance with the guidelines of, and this study was approved by, the animal welfare committee of the Max Planck Society (Regierungspräsidium Karlsruhe, Referat 35, 76247 Karlsruhe), License number 35.9185.81/G-60/02).

Twelve, 4–7 month old, male mice (C57/BL6) were chronically implanted with a hyperdrive –containing three independently adjustable tetrodes and a reference electrode– above the right dorsal CA1. Upon recovery from surgery, tetrodes were adjusted till sharp-wave ripples and multiple single units were detected. The LFP were sampled at 2 kHz and recorded from all tetrodes and the reference electrode with respect to a ground electrode above the cerebellum. The mouse's position and head direction were measured using a CCD camera that tracked the position of two light emitting diodes attached to the hyperdrive. Position data were sampled at 50 Hz with a resolution of 0.25 cm. Position, LFP and spiking data were recorded using the Neuralynx data acquisition system. These data were processed offline using custom software written in Matlab. LFP were filtered using symmetric digital filters and the Hilbert transform was used to obtain the amplitude and phase of each signal in different frequency bands. See Methods S1 for further details. To standardize the amplitude of gamma, the standard deviation of the amplitude of the gamma band signal when the mice were stationary was computed (separately for fast and slow gamma bands) for each electrode in each session. The z-scored amplitude of gamma was obtained by dividing the amplitude of gamma at all speeds by this standard deviation. Thus z = 1 corresponds to 60±1.8 µV and 52±1.6 µV for slow and fast gamma amplitudes respectively.

## Supporting Information

Methods S1 Supplementary materials and methods.
(DOC)

Supplement S1 Data from one electrode each in six different mice (I–VI, mouse label to the left) demonstrating increased slow and fast gamma amplitudes during run compared to stop. Each panel is identical to figure 1C. See figure 1C legend for details.
(DOC)

Supplement S2 Data from the same six mice (I–VI) as in supplement S2, showing speed-dependent, gradual increases in slow and fast gamma amplitude with speed. Each panel is identical to the corresponding panels in figure 2A and 2B. See figure 2A, B legend for details.
(DOC)

Supplement S3 Slow gamma amplitude increased linearly with speed whereas fast gamma amplitude increased logarithmically with speed. The average amplitude of slow gamma in each speed bin was computed for 30 speed bins (figure 2A, supplement S2). A linear fit was made to the average amplitude of slow gamma as a function of speed. The absolute value of the residual error was averaged across all speed bins and divided by the average slow gamma amplitude at all speeds to yield the percentage residual (Solid red line, Supplement S3A). R2 values of the best fit (solid red line, Supplement S3B) were also computed. Small values of the percentage residual error (1.9%) and large values of R2 (median = 0.90) indicate that the linear model is a good fit. However, when the slow gamma amplitude as a function of speed was fit with a logarithmic curve, the percentage residual error was significantly larger (2.9%, dashed line, Supplement S3A), and the R2 value was significantly lower (median = 0.78, dashed red line, Supplement S3B). A similar procedure was followed to compute the goodness of fit of a logarithmic relationship between fast gamma amplitude and speed. In contrast to slow gamma, the percentage residual error was significantly smaller when using a logarithmic fit to fast gamma (1.2%, dashed blue line, Supplement S3C), compared to a linear fit (1.9%, solid blue line, Supplement S3c). Further, *R^2^* values (median = 0.94, dashed blue line, Supplement S3d) were significantly higher with a logarithmic fit for fast gamma than with a linear fit (median = 0.82, solid line, Supplement S3D). Additionally, in panel E the x-axis is the percentage error for a linear fit while the y-axis is for a logarithmic fit. Red dots represent slow gamma and blue dots represent fast gamma for each data set (214 data sets). Most of the red dots were distributed above the diagonal line indicating a better linear fit for slow gamma. On the other hand, most of the blue dots were distributed below the diagonal line indicating a better logarithmic fit for fast gamma. Similar scatter plot was done for *R^2^* values in panel F showing the same results.
(DOC)

Supplement S4 Ensemble averaged speed-dependent cross-frequency coupling (CFC) and differential increase of slow and fast gamma modulation indices. **A**) This figure is similar to figure 2E. Speed-dependent CFC similar to figure 2E was computed for each LFP and averaged across the ensemble of 214 LFPs to obtain this figure. In order to reduce noise, the panel with lowest speed was subtracted from the subsequent panels, leaving only the speed-dependent component. Each vertical panel, for a given speed, shows the cross-frequency coupling between the *amplitude* of the fast (15–300 Hz) signal as a function of the *phase* of the slow (2–20 Hz) signal. Significant cross-frequency coupling is visible between the phase of theta (6–12 Hz) and the amplitude of gamma (20–120 Hz). Consistent with figure 1B, cross-frequency coupling is distinct in the slow and fast gamma bands (bottom panel shows inset for slow gamma). **B**) Modulation index averaged over slow (red line) and fast (blue line) gamma bands and plotted as a function of speed, indicating their differential dependence on speed.
(DOC)

Supplement S5 Theta-phase precession of preferred gamma phase as a function of running speed. Same as in figure 3 but from another six different mice (I–VI, same in Supplement S1, mouse number is to the left of each panel). **A**) Normalized slow gamma amplitude as a function of theta phase at the highest (solid) and lowest (dotted) speeds. **B**) Similar data as A with slow gamma amplitude as a function of theta phase and the logarithm of running speed. **C**) Same as in A for fast gamma. **D**) Same as in B for fast gamma.
(DOC)

Supplement S6 Distribution of preferred theta phase of slow (red) and fast (blue) gamma at low speeds before realignment. At low speeds the mean phase of low gamma was (281±4.1°) and that of high gamma was (269±4.0°).
(DOC)

Supplement S7 Relationship between the magnitude of speed-dependent precession of slow and fast gamma preferred phase of theta. For each LFP the difference in theta preferred phase of slow gamma at the highest minus the lowest speed was computed. A similar difference in theta preferred phase was computed for fast gamma. The speed-dependent change in theta preferred phase of slow and fast gamma were correlated (r = 0.48) with slow gamma showing a significantly greater degree of phase precession than fast gamma.
(DOC)

Supplement S8 A summary of changes in slow and fast gamma amplitude and timing with running speed and theta rhythm. The multiunit activity (green dots) is maximal at the trough of theta (black trace, top and bottom). At low speed (top) the fast gamma amplitude is maximal (blue) just before the slow gamma reaches maximal value (red). At high speed (bottom) the amplitude of both slow and fast gamma, as well as the multiunit firing rate, increase. Further, maximal slow gamma amplitude appears about 15 ms before maximum fast gamma amplitude.
(DOC)
