# Supplementary material for: Speed Controls the Amplitude and Timing of the Hippocampal Gamma Rhythm
Source: PLoS One. 2011 Jun 24;6(6):e21408. doi: 10.1371/journal.pone.0021408 (PMC3123337; doi:10.1371/journal.pone.0021408)
Supplement: Methods S1 — Supplementary materials and methods. (DOC) [file pone.0021408.s001.doc]

**Methods S1**

All experiments were carried out in 12, adult, 4-7 month old, wild-type (C57/BL6) male mice. Mice were housed individually, maintained on a 12-h light/dark cycle and had *ad libitum* access to food and water unless otherwise specified. All experiments were conducted in accordance with the animal welfare guidelines of the Max Planck Society.

**Electrophysiology**: The mice were implanted with hyperdrives above the right dorsal CA1 region of the hippocampus (AP-2.0 mm, L1.5 mm with respect to bregma). Each hyperdrive contained up to 4 independently movable tetrodes and a reference electrode [1,2]. Tetrodes were made of four 15-μm, Teflon coated Nichrome wires (Kanthal, Palm Coast FL), which were twisted and heat-fused together. Two stainless steel screws were implanted over the opposite frontal cortex (for anchoring) and the cerebellum (for ground).

**Behavioral procedures:** One week after surgery, the mice were trained to run back and forth on a linear track for food reward (sweetened milk) located at the opposite ends of the track. The track (length 140 cm, width 3 cm) was made of wood and painted black. Throughout the entire training and further recording the mice were food-restricted and maintained at ~85% of their postoperative *ad libitum* body weights.

**Recording methods:** Throughout the training period, the tetrodes were advanced gradually over the course of many days to place them in the CA1 pyramidal cell layer. The arrival of each tetrode into the hippocampus was recognized by several criteria, including the presence of 100-300 Hz “ripples” in the local field potential (LFP) [3,4,5], the polarity of “sharp waves” in the LFP [6], and the appearance of multiple cells with complex spikes. During recording, the mice were attached to a unitary gain head-stage preamplifier (HS-16; Neuralynx, Tucson, AZ) via a cable suspended on the supporting metal string to minimize an additional load to the animal’s head. Signals were filtered and differentially amplified against the reference electrode by Lynx-8 programmable amplifiers (Neuralynx). Whenever the amplitude of the spike signal exceeded a predetermined threshold, each tetrode channel acquired a 1-ms sample of data at a rate of 32kHz. These spike samples were time-stamped, amplified by a factor of 5,000-10,000 and stored on a personal computer running Cheetah data acquisition software (Neuralynx). Local field potentials (LFP) were recorded from one (of the four) electrodes of each tetrode. The LFP was sampled at 2kHz, amplified 2000 times, and filtered between 0.5-900Hz.

The animal’s position and head direction were measured using an overhead CCD camera (Cohu iDome, USA) that tracked two light-emitting diodes (red and blue) attached to the headstage. Video recording was made with a spatial resolution of 0.25 cm/pixel and a sampling rate of 50Hz. Daily recording sessions consisted of 8-40 laps on the linear track surrounded by rest/sleep sessions (20 min) on an elevated platform.

**Histology**: Following completion of the experiments the mice were deeply anesthetized and electrolesion (200 µA for 4 sec via one channel of each tetrode with respect to the ground screw) was performed to confirm that the tetrodes were in CA1.

**Multi-unit extraction**: All the spikes with peak amplitude greater than 100 μV and peak to valley duration ranging between 0.125ms to 0.375ms were extracted from each tetrode. Only data from tetrodes with more than 1000 such spikes in one session were used. These criteria yielded 141 usable data sets.


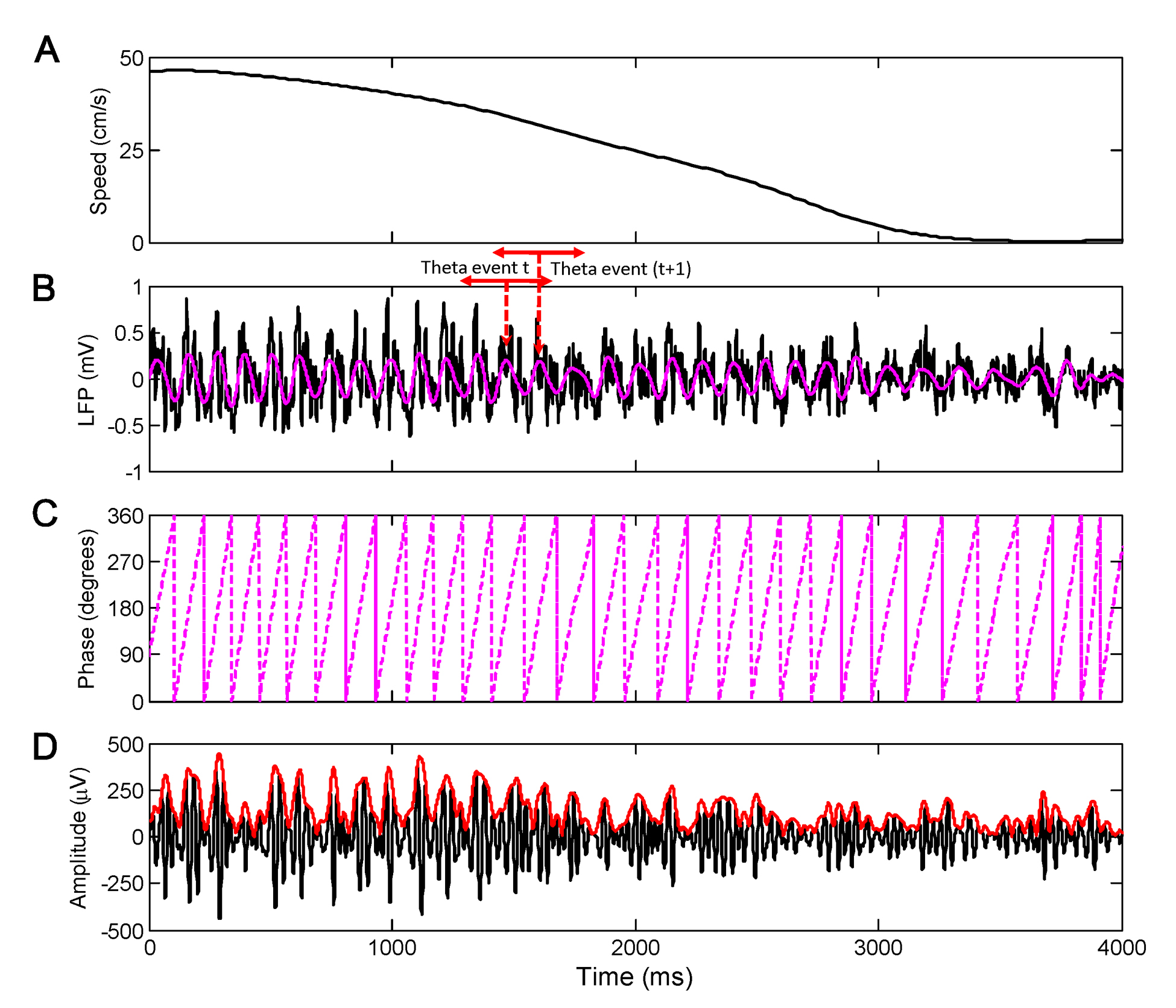


**Figure S1.1:** Speed and spectral analysis. **A**) Speed of one mouse as a function of time. **B**) Broad band (0.5-900Hz) LFP (black) and theta band (6-12Hz) LFP (pink). Each theta event (250ms) was assigned a number (t, t+1, …). **C**) Hilbert transform of the theta band LFP from B. Troughs of theta in B were assigned a phase 0^o^ or 360^o^. **D**) Slow gamma (20-45Hz) LFP (black) and the envelope of the filtered LFP obtained using the Hilbert transform (red).

**Computation of running speed:** Position data were smoothed using a Gaussian kernel of width sigma=120ms to remove noise from the video tracking data. Speed was computed as the first temporal derivative of position (figure S1.1a)

**Power spectral analysis:** LFP were filtered in appropriate bands using a two-way least squares FIR filter. The LFP spectrum was computed using the multi-taper method from the Chronux open source MATLAB toolbox (<http://www.chronux.org/>). For figure 1A, the spectrogram was analyzed for one LFP trace from one example session. The spectrum was calculated in 2s epochs, and was averaged separately for data when the animal was running or immobile. Mice alternated between immobility and run in each trial. Spectral power was computed in a 2s wide window placed at the center of each run or immobile epoch. The spectral power at each frequency was averaged across all the 29 run and stop epochs in the calculation. Confidence intervals were estimated using the jackknife method.

For figure 1B, the change of spectral power was estimated between the run and stop spectra calculated in figure 1A using:

$$Percentage\text{ }change\text{ =100}\times\frac{S_{run}-S_{imm}}{S_{imm}}$$

Where *S_run_* is the run spectrum and *S_imm_* is the stop spectrum. The result was averaged across 214 LFP traces obtained in 63 sessions from 12 mice. The shaded region indicates the standard error of the mean in this and all figures.

**Theta event based analysis of gamma amplitude:** Each LFP was first filtered in the theta band (6-12Hz) and then Hilbert transformed to locate the peak for each individual theta cycle. Theta peaks were assigned a phase of 180 degrees and the relative phase of theta was computed within each theta cycle. The entire data set was then labeled with a series of theta events centered on theta peaks with a 250ms window length (~50% overlap with both adjacent theta events, figure S1.1B). Slow (fast) gamma amplitudes were obtained by filtering the LFP in 20-45Hz (45-120Hz) bands and was computed from the absolute value of the analytic signal. After this labeling was done, the average speed, and fast and slow gamma amplitudes were calculated for each theta event. Figures 2A-B were obtained by plotting speed vs slow or fast gamma amplitude for all the theta events from one data set.

**Speed dependent cross frequency coupling (CFC):** For ease of depiction, only six speed bins were used in this analysis (figure 2E). The first bin was for immobility with speeds between 0 - 0.5cm/s. The remaining five equally spaced bins were generated between 2cm/s to the top 99% speed for all the theta events. There was no overlap between neighboring speed bins. The range of frequencies used for the amplitude-frequency sweep was from 15-300Hz, with a step size of 2Hz, and bandwidth of 4Hz, while the range used for phase-frequency sweeps was from 2-20Hz, with a step size of 1Hz, and a bandwidth of 2Hz. To accommodate these lower frequencies, CFC and speed were computed in coarser, 500ms wide windows. The results were qualitatively insensitive to these choices of parameters.

For each amplitude and phase frequency pair in a given speed bin, the data were grouped into 60 equally spaced phase bins spanning 0-360^o^ based on their phase value, and the mean amplitude of the high frequency signal *f_amp_* was calculated for each low frequency phase bin *f_phase_* for a given speed bin *S*. Using these, the Shannon entropy (*H*) of cross-frequency coupling and its modulation index (*MI*) were calculated [7] as:

$$H(f_{amp},f_{phase},S)=-\sum_{j=1}^{N} P_{j}(f_{amp},f_{phase},S)\log[P_{j}(f_{amp},f_{phase},S)]$$

$$MI(f_{amp},f_{phase},S)=\frac{\log(N)-H}{\log(N)}$$

Where *P(j)* is the normalized amplitude of the high frequency signal in each low frequency phase bin *j*, and *N* is the number of phase bins (60). Modulation Index *MI* is normalized using *log(N)* and defined this way because the highest value of *H* is *log(N)*, occurring for a uniform distribution of phases, indicative of minimal modulation. Figure 2E and supplement S4 were obtained using this procedure.

**
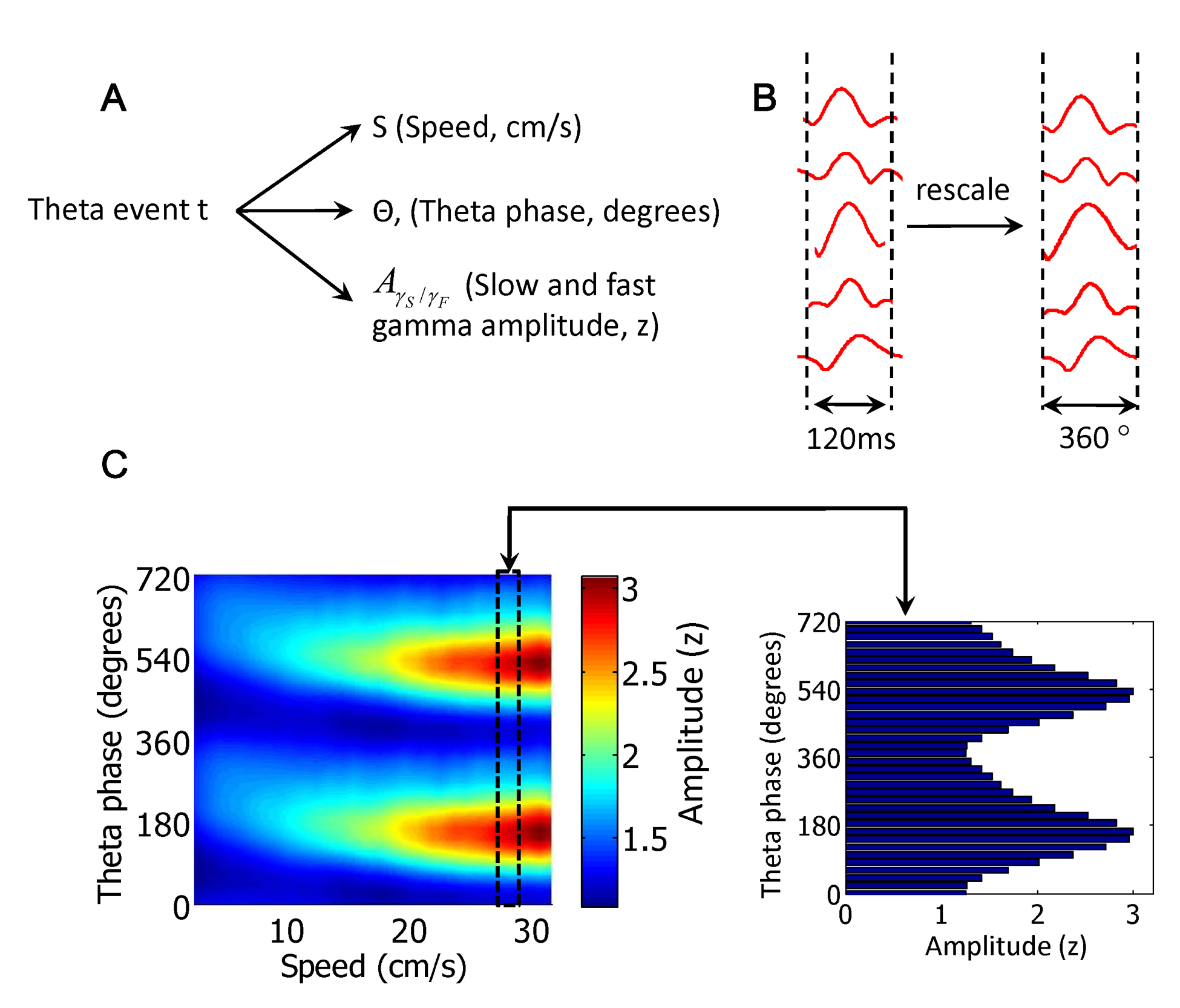
**

**Figure S1.2:** Velo-temporal receptive fields for speed (VTRF). **A**) Speed S, theta phase and slow/fast gamma amplitude were calculated within each theta event *t*. **B**) Slow/fast gamma amplitude envelope for each theta event was rescaled to the same length spanning 0-360^o^ of the theta cycle. **C**) The rescaled gamma amplitude envelopes were grouped into different speed bins and the average gamma amplitude value at each theta phase was obtained for each speed bin (right panel). Slow gamma amplitude (colorbar, z-scored units) as a function of speed and theta phase (left).

**Computation of the joint influence of speed and theta phase on gamma amplitude and hippocampal velo-temporal receptive fields (VTRF):** Since cross-frequency coupling was mostly restricted to theta-gamma coupling (see above), subsequent analysis focused on the fine temporal structure of theta-gamma CFC as a function of speed. LFPs were filtered in the theta (6-12Hz) band and theta phase was computed as described above. The amplitude of the LFP in the slow and fast gamma bands was also computed as detailed above. To obtain finer temporal resolution, theta phase was separated into 120 phase bins at each speed. To obtain finer resolution of speed, thirty speed bins with 80% overlap between adjacent speed bins were used. Both theta and gamma data were sorted into one of the thirty speed bins as described above (figure S1.2A, B). The reason to use a fixed number of speed bins rather than a fixed speed bin width was to allow ensemble average across different data sets where mice spanned different ranges of speed. The mean gamma amplitude within each speed-phase bin was calculated separately for slow and fast gamma. This procedure yielded a 2D picture (figure S1.2C) that showed the joint modulation of slow (fast) gamma amplitude by running speed and theta phase, called the velo-temporal receptive field for speed (VTRF; figure 2F-G).

**Computation of speed-dependent shift of preferred theta phase for slow and fast gamma:** This was obtained by first calculating the preferred phase of slow (or fast) gamma amplitude in each speed bin from the previously calculated VTRF. Circular analysis was used to calculate this preferred phase, given by:

$$\theta^{\gamma_{S}/\gamma_{F}}(S)=\arg(\sum_{j=1}^{N} P_{j}^{\gamma_{S}/\gamma_{F}}(S)\times e^{i\theta_{j}})$$

where *N* is the number of phase bins, *S* is the speed, and *γ_S_* and *γ_F_* denote the slow and fast gamma bands. $P_{j}^{\gamma_{S}/\gamma_{F}}$is the mean gamma amplitude in each phase bin at a certain speed *S* for slow or fast gamma, and $\theta_{j}$is the value of phase at the center of each phase bin *j*.

**Normalization of gamma amplitude across data:** To calculate the dependence of gamma amplitude on speed and theta phase, averaged across data sets from different tetrodes, sessions and animals, gamma amplitude had to be standardized across data sets. Since different animals ran at different speeds, and gamma amplitude varied across electrodes and sessions, simple averaging of gamma amplitude across all speeds would generate variable results. Hence, the filtered slow (or fast) gamma amplitudes were divided by the standard deviation of the gamma amplitude during times when the animals were immobile. This made the mean amplitude envelope of slow (or fast) gamma during immobility close to one for all the data sets. This normalization of gamma amplitude was done separately for fast and slow gamma and for each LFP. As a result, all the speed-dependent gamma amplitude traces (figures 2-4, S3-S6) start close to unity at the lowest speed, which allows unbiased comparison and averaging across diverse data.

**Realignment of gamma phase precession across data:** A similar procedure was followed to compute the ensemble averaged velo-temporal receptive fields for speed (VTRF) (figure 3C, G and figure 4A). The preferred theta-phase of slow (or fast) gamma varied across electrodes (supplement S6). To remove the variability introduced by this, a procedure similar to that used to compute the ensemble averaged phase precession of spikes as a function of position was used. Specifically, since the average preferred phase of slow and fast gamma was around 270^o^ (supplement S6), the preferred theta-phase of slow gamma in each dataset was reset to 270^o^ for all LFPs. Across the ensemble of data, the average phase offset thus introduced was small, (-28.95^o^±3.58^o^). Speed-dependent change in the slow gamma’s preferred theta-phase was then averaged across all the data to obtain figures 3C, 4A and supplement S5.

Notably, the exact amount of constant phase realignment done for slow gamma for each LFP trace was also carried out for fast gamma phase for that LFP. This ensured that the phase difference between slow and fast gamma theta-phases was unchanged in each electrode.

**Gamma phase locking of spike probability:** The Hilbert transform was applied to the LFP in the slow and fast gamma bands to obtain their respective phases. The slow and fast gamma phase for each spike at the time of occurrence was computed. The instantaneous slow and fast gamma cycles where each spike occurred were identified and the slow and fast gamma amplitudes at the peak and the trough were assigned to each spike. The gamma amplitude was normalized with the same method described above to facilitate the comparison between different data sets. Only the spikes with slow or fast gamma amplitude greater than 1.5z both at the peak and trough were used in the slow or fast gamma phase locking analysis.

Preferred phase and modulation index of spike phase locking were computed separately and similarly for slow and fast gamma as follows. Spikes were sorted into N phase bins according to their slow (or fast) gamma phase. The number of spikes in each bin was divided by the total number of spikes to yield a spike phase probability distribution (whose sum is unity). The preferred gamma phase φ and gamma modulation index GMI were defined as:

$$GMI=abs(\sum_{j=1}^{N} p_{j}e^{i\theta_{j}})$$

where *N* is the number of phase bins, and *p_j_* is the probability of spikes occurring in a phase bin *θ_j_*. These definitions have the advantage that the modulation index is independent of the mean firing rate and the number of phase bins.

**Supplementary References:**

1. McNaughton BL, O'Keefe J, Barnes CA (1983) The stereotrode: a new technique for simultaneous isolation of several single units in the central nervous system from multiple unit records. J Neurosci Methods 8: 391-397.

2. Wilson MA, McNaughton BL (1993) Dynamics of the hippocampal ensemble code for space. Science 261: 1055-1058.

3. O'Keefe J (1976) Place units in the hippocampus of the freely moving rat. Exp Neurol 51: 78-109.

4. O'Keefe J, Conway DH (1978) Hippocampal place units in the freely moving rat: why they fire where they fire. Exp Brain Res 31: 573-590.

5. Buzsaki G, Horvath Z, Urioste R, Hetke J, Wise K (1992) High-frequency network oscillation in the hippocampus. Science 256: 1025-1027.

6. Buzsaki G, Czopf J, Kondakor I, Kellenyi L (1986) Laminar distribution of hippocampal rhythmic slow activity (RSA) in the behaving rat: current-source density analysis, effects of urethane and atropine. Brain Res 365: 125-137.

7. Tort AB, Komorowski R, Eichenbaum H, Kopell N Measuring phase-amplitude coupling between neuronal oscillations of different frequencies. J Neurophysiol 104: 1195-1210.
