# Supplementary figures and images for: Speed Controls the Amplitude and Timing of the Hippocampal Gamma Rhythm
Source: PLoS One. 2011 Jun 24;6(6):e21408. doi: 10.1371/journal.pone.0021408 (PMC3123337; doi:10.1371/journal.pone.0021408)

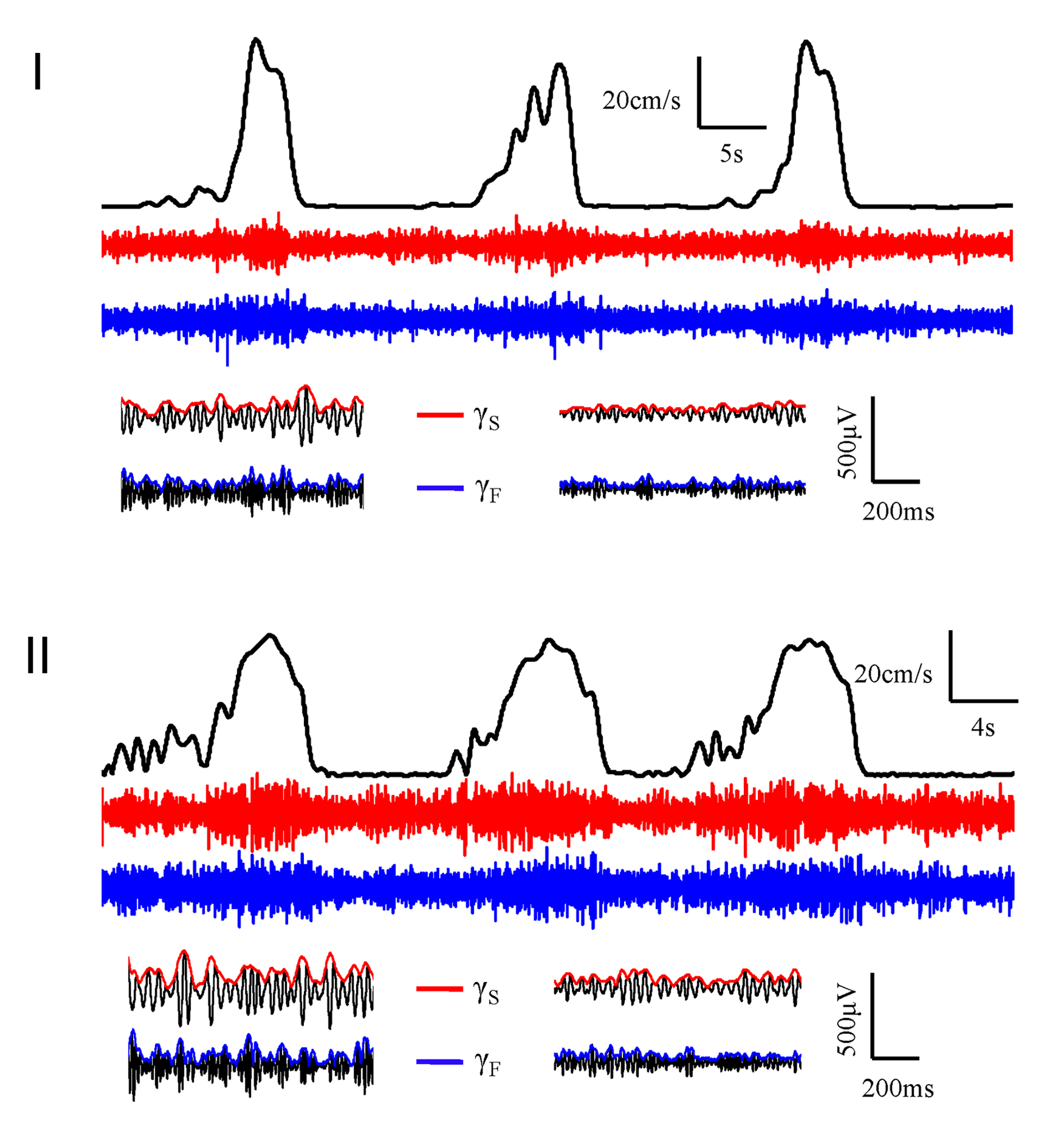


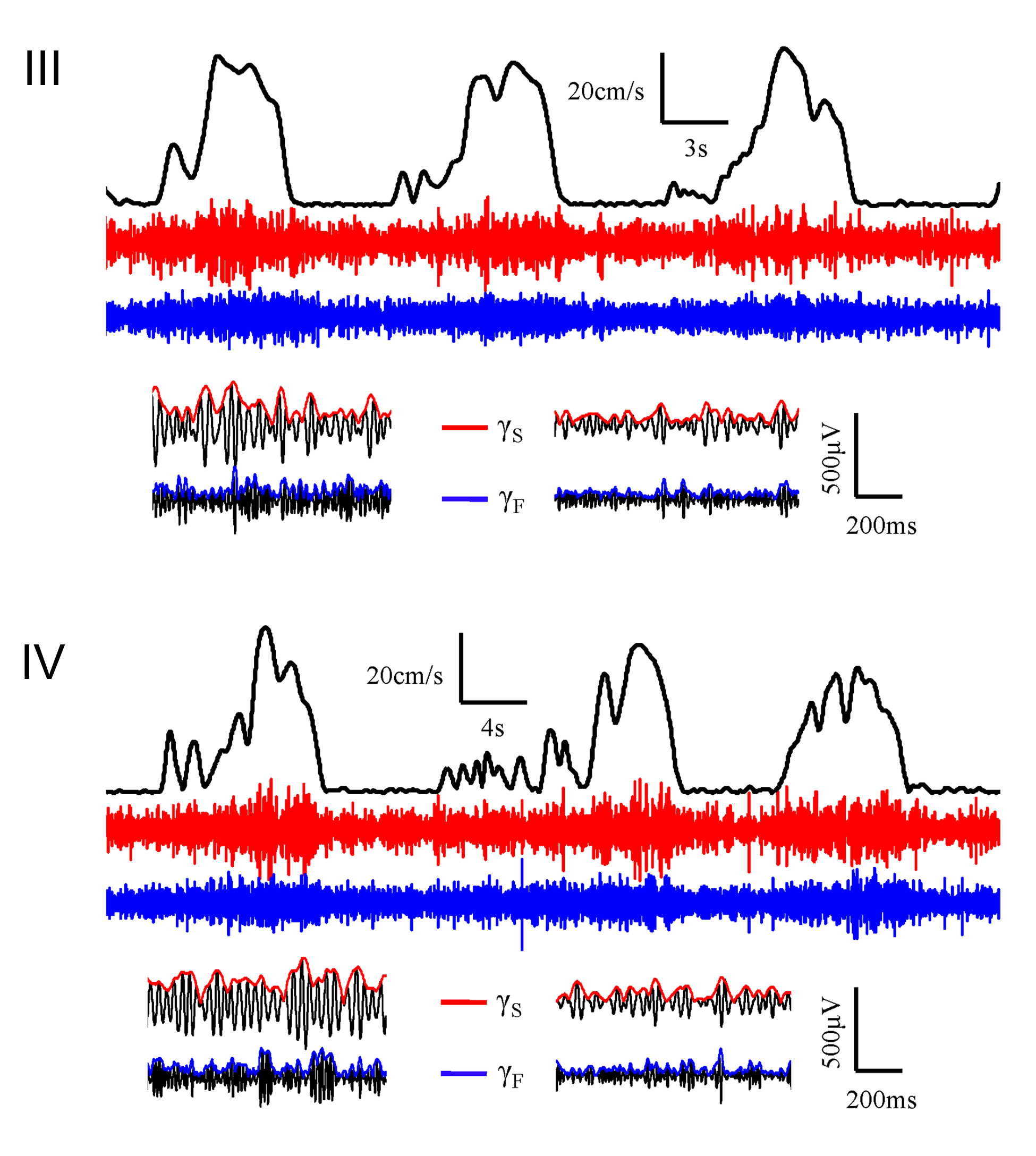


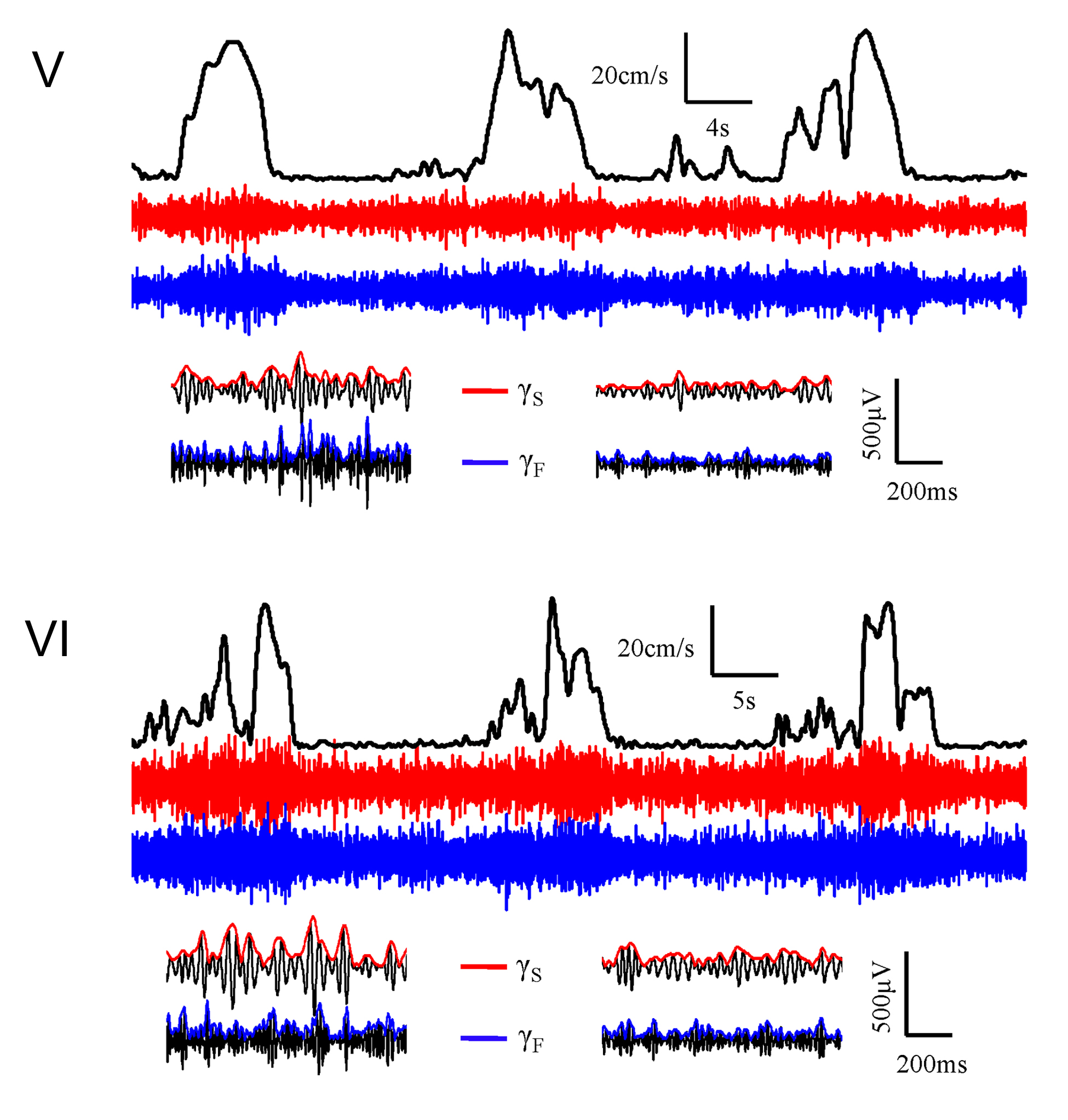

Supplement: Supplement S1 — Data from one electrode each in six different mice (I–VI, mouse label to the left) demonstrating increased slow and fast gamma amplitudes during run compared to stop. Each panel is identical to figure 1C. See figure 1C legend for details. (DOC) [file pone.0021408.s002.doc]

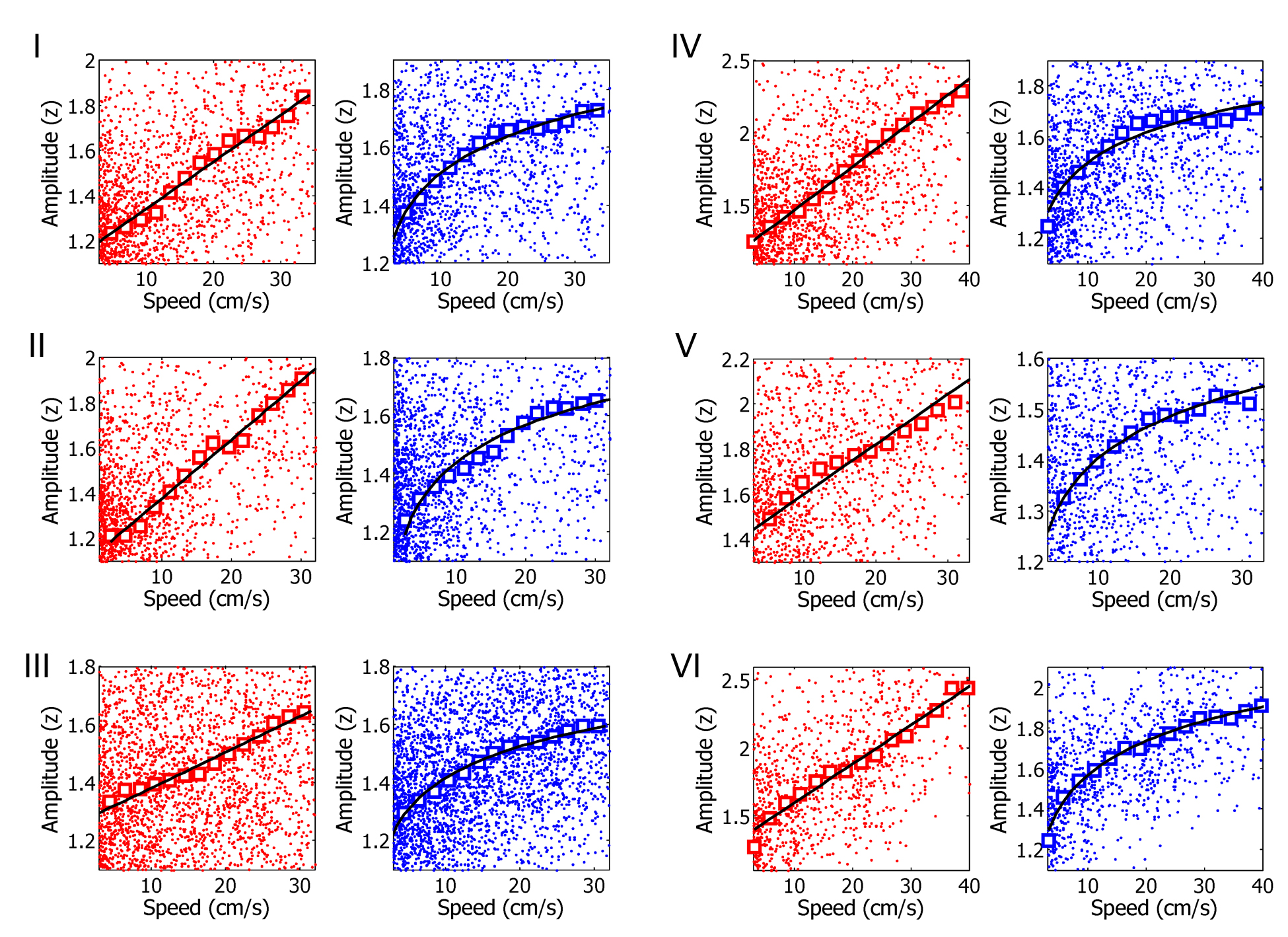

Supplement: Supplement S2 — Data from the same six mice (I–VI) as in supplement S2, showing speed-dependent, gradual increases in slow and fast gamma amplitude with speed. Each panel is identical to the corresponding panels in figure 2A and 2B. See figure 2A, B legend for details. (DOC) [file pone.0021408.s003.doc]

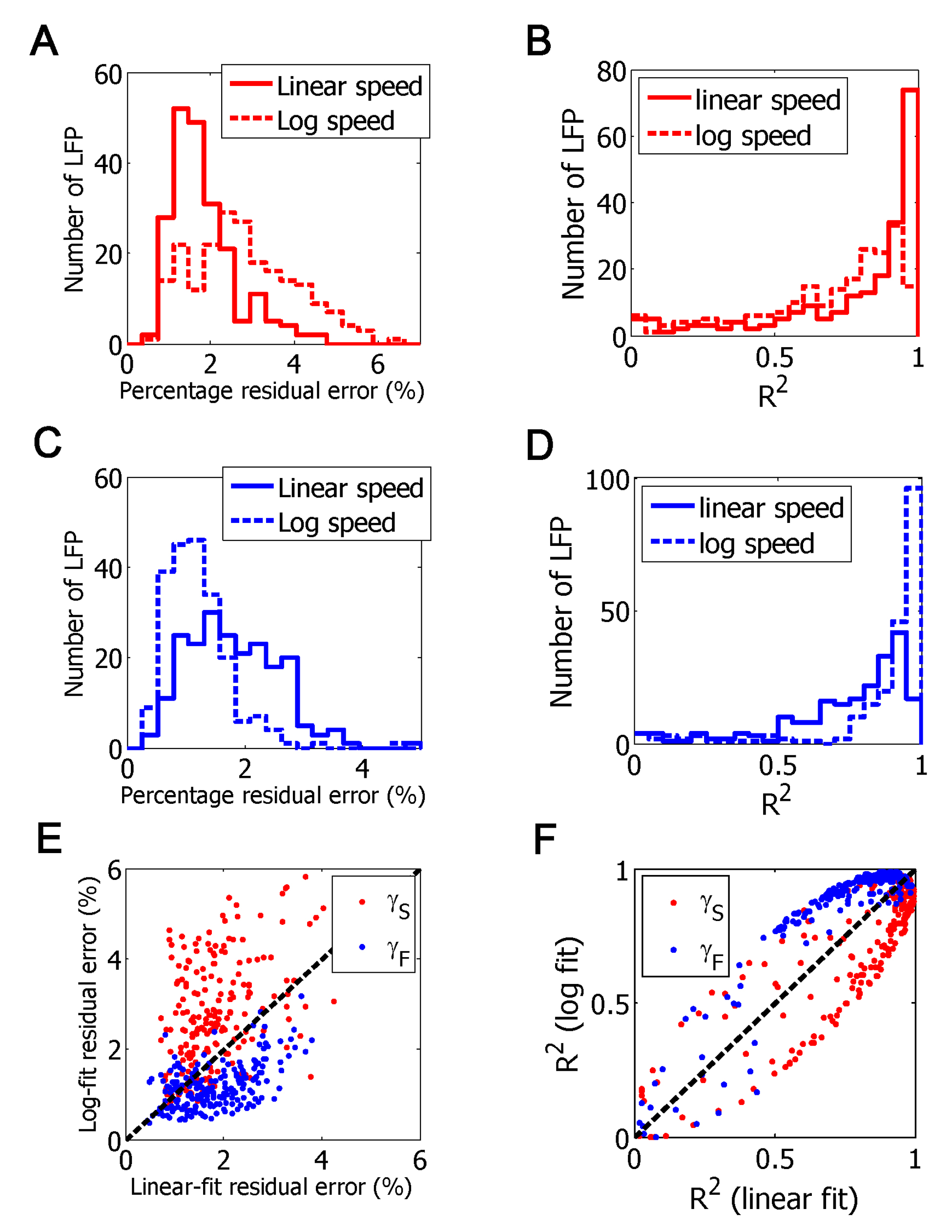

Supplement: Supplement S3 — Slow gamma amplitude increased linearly with speed whereas fast gamma amplitude increased logarithmically with speed. The average amplitude of slow gamma in each speed bin was computed for 30 speed bins (figure 2A, supplement S2). A linear fit was made to the average amplitude of slow gamma as a function of speed. The absolute value of the residual error was averaged across all speed bins and divided by the average slow gamma amplitude at all speeds to yield the percentage residual (Solid red line, Supplement S3A). R2 values of the best fit (solid red line, Supplement S3B) were also computed. Small values of the percentage residual error (1.9%) and large values of R2 (median = 0.90) indicate that the linear model is a good fit. However, when the slow gamma amplitude as a function of speed was fit with a logarithmic curve, the percentage residual error was significantly larger (2.9%, dashed line, Supplement S3A), and the R2 value was significantly lower (median = 0.78, dashed red line, Supplement S3B). A similar procedure was followed to compute the goodness of fit of a logarithmic relationship between fast gamma amplitude and speed. In contrast to slow gamma, the percentage residual error was significantly smaller when using a logarithmic fit to fast gamma (1.2%, dashed blue line, Supplement S3C), compared to a linear fit (1.9%, solid blue line, Supplement S3c). Further, R2 values (median = 0.94, dashed blue line, Supplement S3d) were significantly higher with a logarithmic fit for fast gamma than with a linear fit (median = 0.82, solid line, Supplement S3D). Additionally, in panel E the x-axis is the percentage error for a linear fit while the y-axis is for a logarithmic fit. Red dots represent slow gamma and blue dots represent fast gamma for each data set (214 data sets). Most of the red dots were distributed above the diagonal line indicating a better linear fit for slow gamma. On the other hand, most of the blue dots were distributed below the diagonal line ind [file pone.0021408.s004.doc]

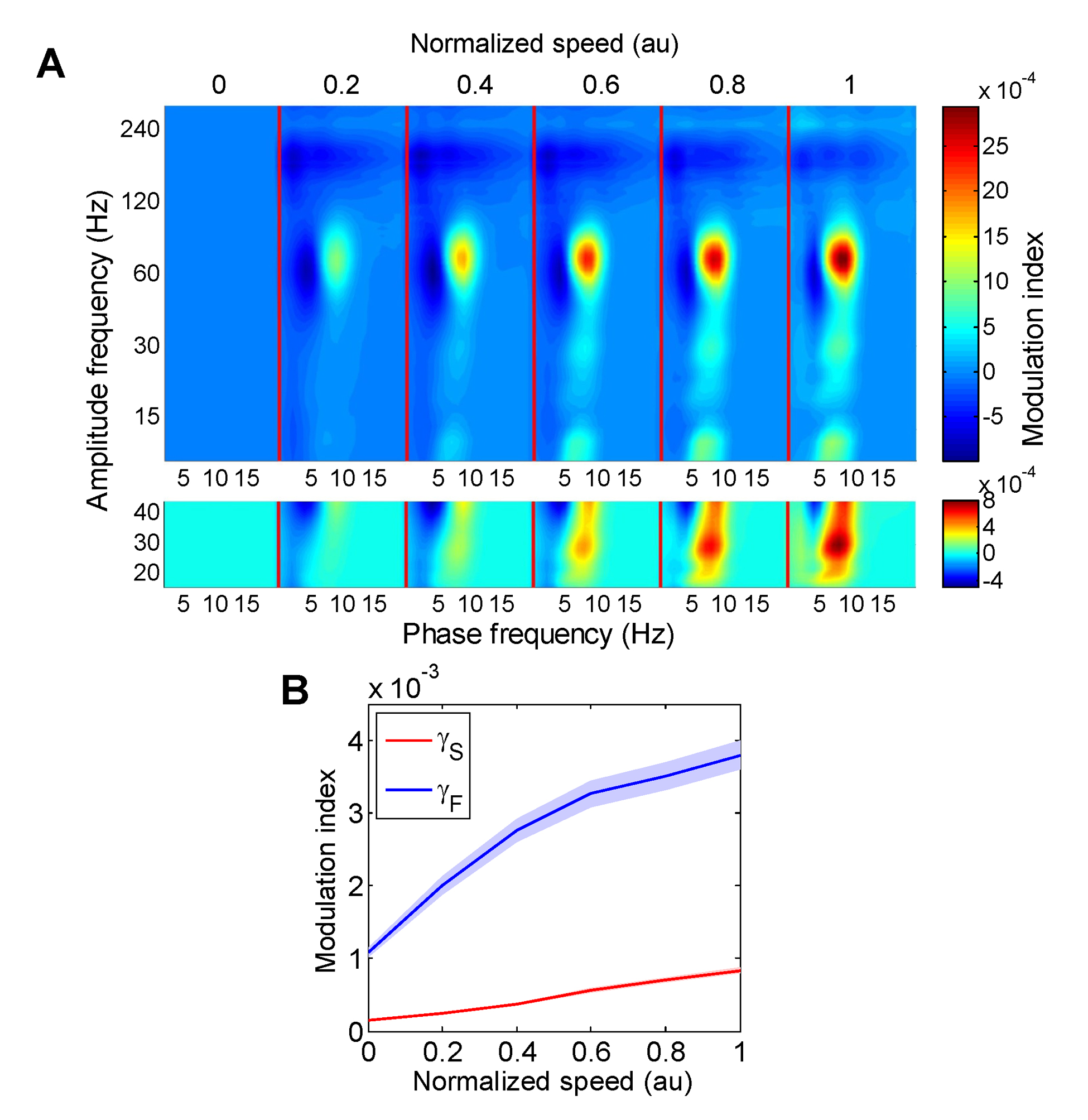

Supplement: Supplement S4 — Ensemble averaged speed-dependent cross-frequency coupling (CFC) and differential increase of slow and fast gamma modulation indices. A) This figure is similar to figure 2E. Speed-dependent CFC similar to figure 2E was computed for each LFP and averaged across the ensemble of 214 LFPs to obtain this figure. In order to reduce noise, the panel with lowest speed was subtracted from the subsequent panels, leaving only the speed-dependent component. Each vertical panel, for a given speed, shows the cross-frequency coupling between the amplitude of the fast (15–300 Hz) signal as a function of the phase of the slow (2–20 Hz) signal. Significant cross-frequency coupling is visible between the phase of theta (6–12 Hz) and the amplitude of gamma (20–120 Hz). Consistent with figure 1B, cross-frequency coupling is distinct in the slow and fast gamma bands (bottom panel shows inset for slow gamma). B) Modulation index averaged over slow (red line) and fast (blue line) gamma bands and plotted as a function of speed, indicating their differential dependence on speed. (DOC) [file pone.0021408.s005.doc]

**
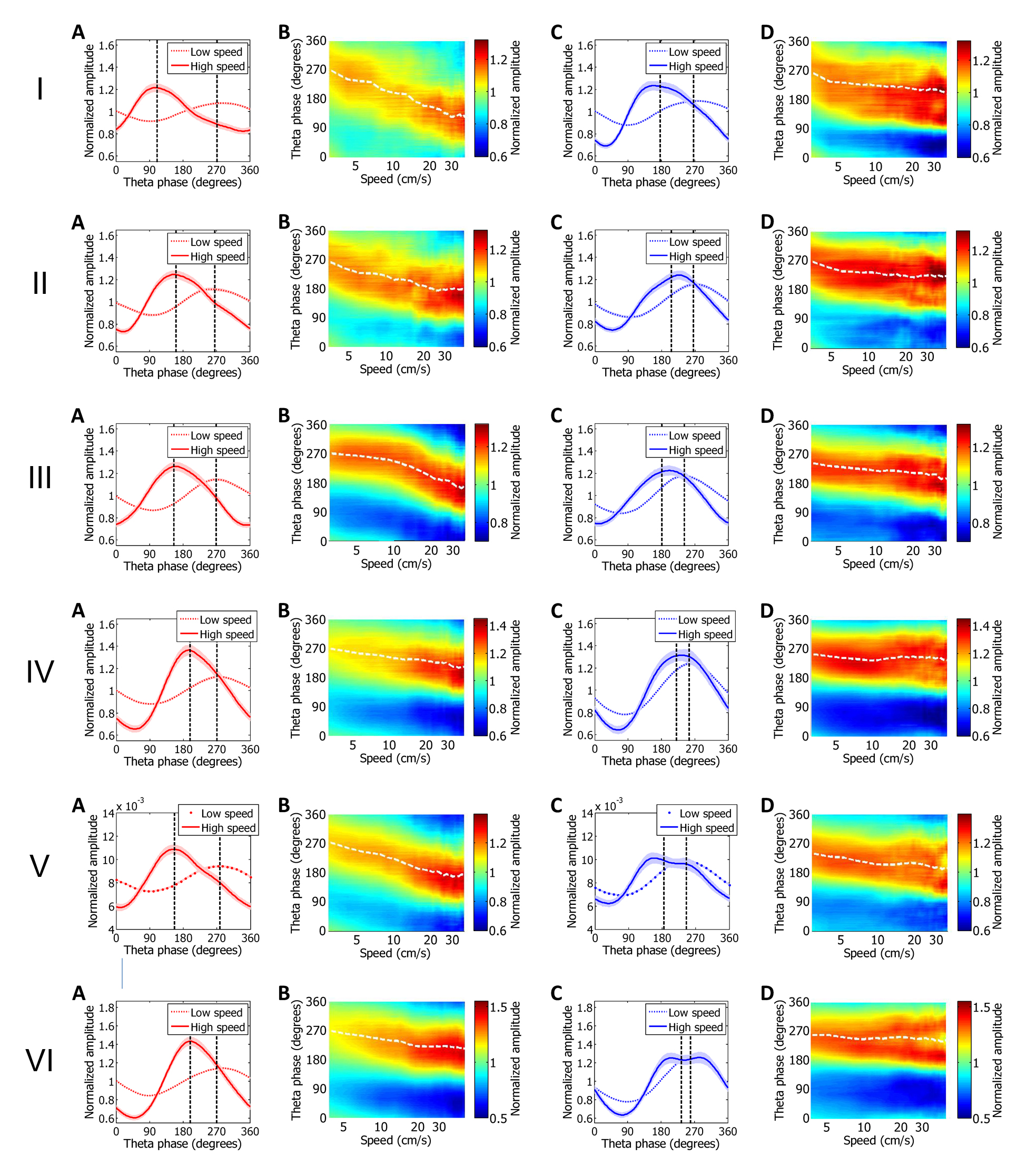
**

Supplement: Supplement S5 — Theta-phase precession of preferred gamma phase as a function of running speed. Same as in figure 3 but from another six different mice (I–VI, same in Supplement S1, mouse number is to the left of each panel). A) Normalized slow gamma amplitude as a function of theta phase at the highest (solid) and lowest (dotted) speeds. B) Similar data as A with slow gamma amplitude as a function of theta phase and the logarithm of running speed. C) Same as in A for fast gamma. D) Same as in B for fast gamma. (DOC) [file pone.0021408.s006.doc]

**
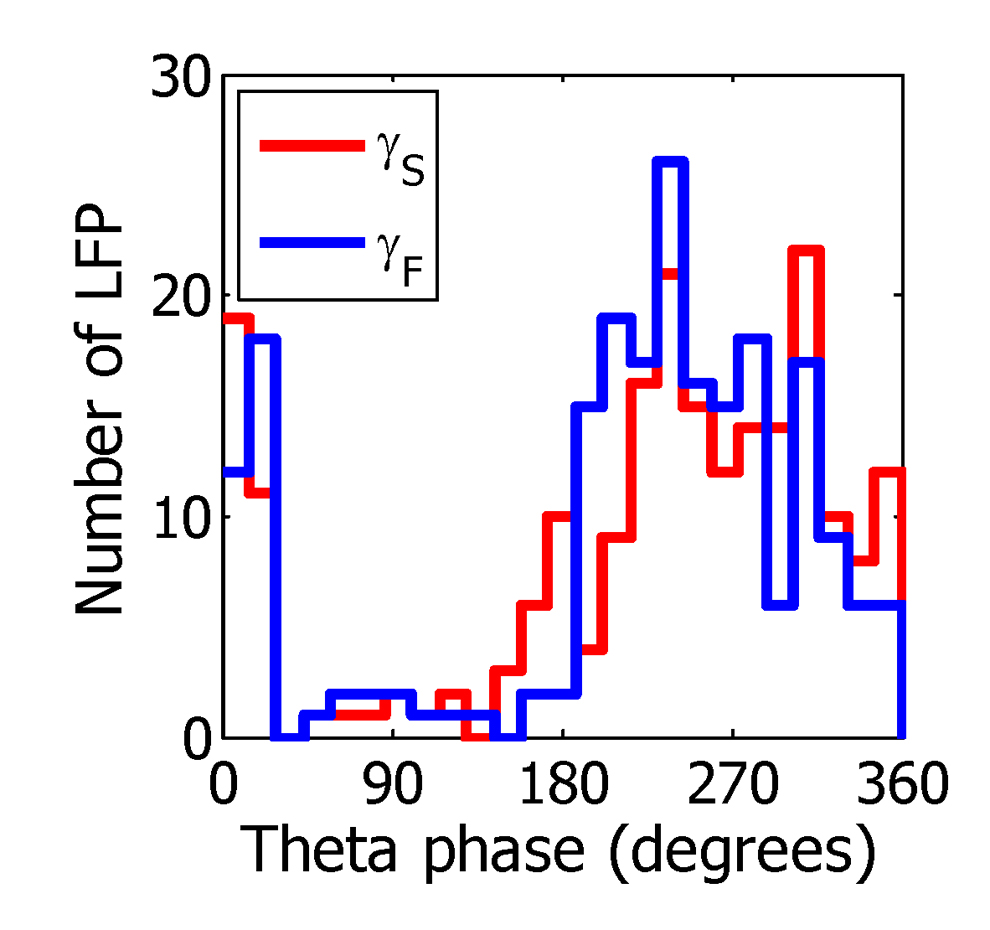
**

Supplement: Supplement S6 — Distribution of preferred theta phase of slow (red) and fast (blue) gamma at low speeds before realignment. At low speeds the mean phase of low gamma was (281±4.1°) and that of high gamma was (269±4.0°). (DOC) [file pone.0021408.s007.doc]

**
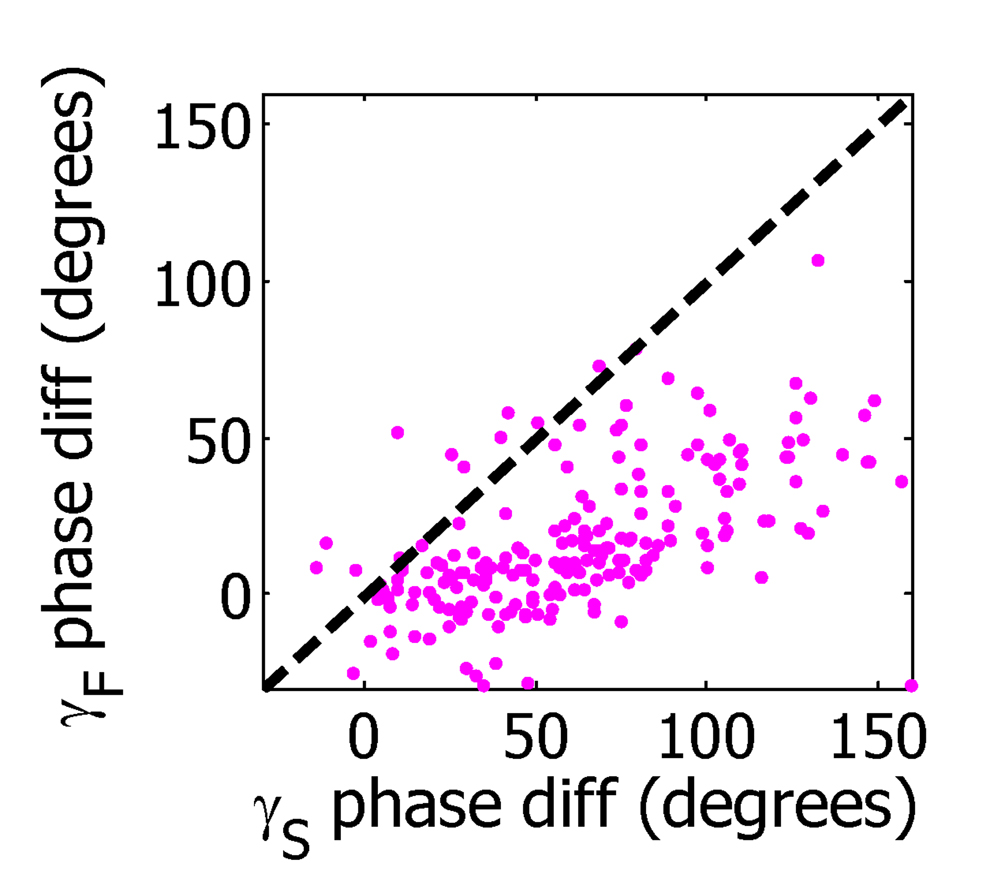
**

Supplement: Supplement S7 — Relationship between the magnitude of speed-dependent precession of slow and fast gamma preferred phase of theta. For each LFP the difference in theta preferred phase of slow gamma at the highest minus the lowest speed was computed. A similar difference in theta preferred phase was computed for fast gamma. The speed-dependent change in theta preferred phase of slow and fast gamma were correlated (r = 0.48) with slow gamma showing a significantly greater degree of phase precession than fast gamma. (DOC) [file pone.0021408.s008.doc]

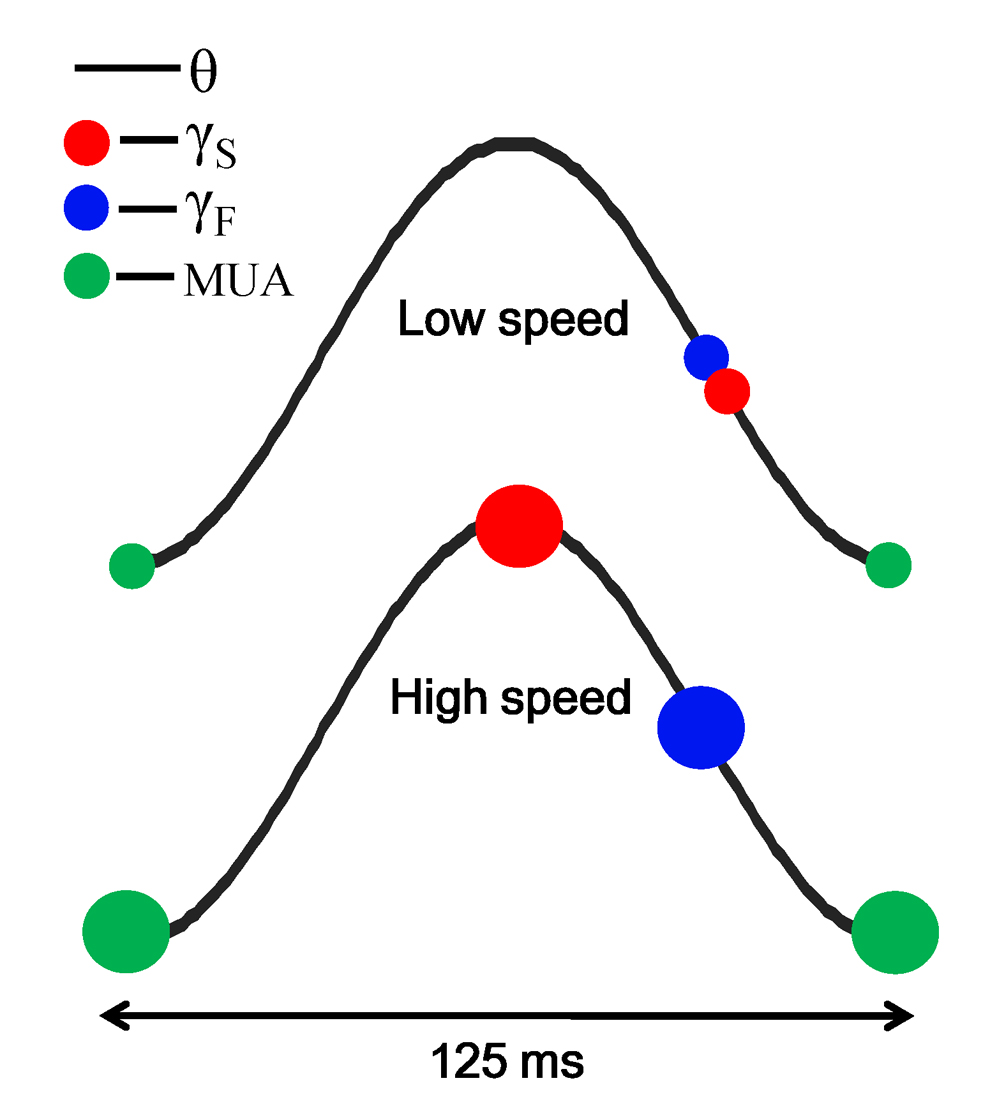

Supplement: Supplement S8 — A summary of changes in slow and fast gamma amplitude and timing with running speed and theta rhythm. The multiunit activity (green dots) is maximal at the trough of theta (black trace, top and bottom). At low speed (top) the fast gamma amplitude is maximal (blue) just before the slow gamma reaches maximal value (red). At high speed (bottom) the amplitude of both slow and fast gamma, as well as the multiunit firing rate, increase. Further, maximal slow gamma amplitude appears about 15 ms before maximum fast gamma amplitude. (DOC) [file pone.0021408.s009.doc]
